# Structural Design and Motion Characteristics Analysis of the Inner Wall Grinding Robot for PCCP Pipes

**DOI:** 10.3390/s26030818

**Published:** 2026-01-26

**Authors:** Yanping Cui, Ruitian Sun, Zhe Wu, Xingwei Ge, Yachao Cao

**Affiliations:** School of Mechanical Engineering, Hebei University of Science and Technology, Shijiazhuang 050018, China; cuiyp@hebust.edu.cn (Y.C.); iknow_66@163.com (R.S.); gexingwei@163.com (X.G.); yachao.cao@hebust.edu.cn (Y.C.)

**Keywords:** PCCP pipeline, pipeline inner wall polishing robot, Adams, multibody dynamics simulation, pipeline passability

## Abstract

Internal wall grinding of pipes constitutes a critical pretreatment procedure in the anti-corrosion repair operations of Prestressed Concrete Cylinder Pipes (PCCP). To address the limitations of low efficiency and poor safety associated with traditional manual internal wall grinding in PCCP anti-corrosion repair, this study presents the design of a support-wheel-type internal wall grinding robot for pipes. The robot’s structure comprises a walking support module and a grinding module: the walking module employs four sets of circumferentially equally spaced (90° apart) independent-support wheel groups. Through an active–passive collaborative adaptation mechanism regulated by pre-tensioned springs and lead screws, the robot can dynamically conform to the inner wall of the pipe, ensuring stable locomotion. The grinding module is connected to the walking module via a slewing bearing and is equipped with three roller-type steel brushes. During operation, the grinding module revolves around the pipe axis, while the roller brushes rotate simultaneously, generating a composite three-helix grinding trajectory. Mathematical models for the robot’s obstacle negotiation, bend traversal, and grinding motion were established, and multi-body dynamics simulations were conducted using ADAMS for verification. Additionally, a physical prototype was developed to perform basic functional tests. The results demonstrate that the robot’s motion characteristics are highly consistent with theoretical analyses, exhibiting stable and reliable operation, excellent pipe traversability, and robust driving capability, thus meeting the requirements for internal wall grinding of PCCP pipes.

## 1. Introduction

### 1.1. Research Background and Significance

The water supply assurance project for the Langzhuo Canal, a supporting project of the South-to-North Water Diversion in Hebei Province, is a crucial component in the allocation of water resources in North China, and its stable operation is of paramount importance [1,2]. In the South-to-North Water Diversion Project, prestressed concrete cylinder pipes (PCCP) are widely used due to their excellent durability and load-bearing capacity [3]. However, over time, the inner walls of these pipes face increasing issues such as corrosion, scaling, and sediment accumulation, which seriously affect water conveyance efficiency and pipeline safety. The pipelines requiring repair cannot be directly excavated and replaced due to the presence of existing structures on the surface; traditional cleaning methods are not only inefficient but also pose safety hazards, making them inadequate to meet the urgent maintenance needs of current projects. Therefore, developing an efficient, safe, and suitable robot for grinding and cleaning the inner walls of large-diameter PCCP pipelines is especially important.

Although significant advances have been made in pipeline robot technology, the current robotic systems still face challenges in grinding operations for the inner walls of large-diameter pipelines, specifically, insufficient efficiency and limited adaptability to diameter changes. To address this technical bottleneck, this study systematically establishes a mathematical model for the motion of the pipeline robot on the pipe inner wall and conducts a detailed theoretical analysis. The model is then verified through simulation using ADAMS to ensure the accuracy of the theoretical analysis. Based on this theoretical foundation and simulation results, this study further designs and manufactures a corresponding physical prototype, aiming to achieve efficient and flexible grinding operations on the inner walls of large-diameter pipelines.

### 1.2. Analysis of Existing Research Findings

Prestressed Concrete Cylinder Pipes (PCCP) are widely used in major water conveyance projects. After long-term service, their inner walls are prone to deterioration. Prior to repair, polishing treatment is necessary to ensure the quality of subsequent restoration [4,5,6,7,8,9,10]. Currently, PCCP polishing technology has developed in a diversified trend; however, there is a distinct research gap in specialized automated polishing equipment for large-scale PCCP, making it difficult to meet practical engineering needs.

From the perspective of technical development, PCCP polishing technology has gone through four stages: Traditional mechanical polishing relies on manual handheld equipment, which is only suitable for small-scale repairs and has problems such as low efficiency, uneven quality, and potential safety hazards; Semi-automatic special polishing equipment realizes localized automated polishing for manufacturing and installation needs, improving efficiency and precision but with limited application scenarios; New environmentally friendly polishing, represented by abrasive water jet technology, solves the problem of dust pollution but is difficult to promote on a large scale due to high costs. Although small-scale automated robots have demonstrated value in polishing medium and small-diameter pipelines, they cannot be adapted to the needs of large-scale PCCP [11,12,13]. Further, from the classification of pipeline robot solutions, existing equipment can be divided by mobile configuration and functional positioning: In terms of mobile configuration, wheeled robots move quickly but have poor adaptability to rough inner walls and unstable posture in large-scale pipelines; articulated robots can achieve variable diameter and steering but have complex structures and weak load-bearing capacity; tracked robots have stable support and good adaptability to rough inner walls but limited variable diameter range, all of which fail to meet the wide-range diameter variation requirements of large-scale PCCP. In terms of functional positioning, detection-type robots are technically mature but lack polishing capability; polishing-type robots are only suitable for medium- and small-diameter pipelines and cannot meet the full-section polishing and variable diameter requirements of large-scale PCCP simultaneously [14,15,16,17].

Overall, existing technologies have significant core limitations for polishing large-scale PCCP: Firstly, insufficient equipment adaptability, failing to meet the requirements of large-size variation and full-section polishing with poor support stability; Secondly, low functional integration, with separated detection and polishing functions leading to low operation and maintenance efficiency; thirdly, inadequate stability and intelligence level, lacking adaptive adjustment capability for complex working conditions, and polishing schemes relying on pipeline rotation are infeasible, making it difficult to balance efficiency and environmental protection; fourthly, the research focus is biased towards medium- and small-diameter pipelines, and there is a lack of mature schemes for full-section automated polishing of large-scale PCCP.

To address the full-section, high-precision, and automated polishing requirements of large-scale PCCP, this paper proposes a research and development scheme for a specialized polishing robot. This scheme will focus on breaking through the bottlenecks of adaptability and stability of existing equipment. By designing a large-stroke variable-diameter support mechanism and an adaptive polishing head and integrating visual positioning and force feedback control functions, intelligent adjustment of the polishing process will be achieved. Meanwhile, it will balance operational efficiency and environmental protection, aiming to fill the technical gap in automated polishing of the inner wall of large-scale PCCP and provide reliable technical support for pipeline repair projects.

### 1.3. Principal Contents of This Dissertation

A novel “walking/support + rotary grinding” dual-module integrated configuration is proposed for large-diameter PCCP inner-wall operations.

A four-support force-coupling model quantifies traction, cable-drag,, and grinding disturbances on robot attitude, yielding a reusable design-evaluation framework.

Geometric obstacle-crossing limits, elbow “transition–rotation” two-phase kinematics, and full-coverage grinding path models are provided, laying the theoretical ground for trajectory planning and intelligent control.

ADAMS simulations and PLC-based prototype tests achieve forward/backward travel, radial adaptation, and bidirectional grinding, confirming the scheme’s feasibility.

## 2. Structural Design and Operating Principle

### 2.1. Operational Requirements Analysis and Overall Structural Design

**Figure 1:**
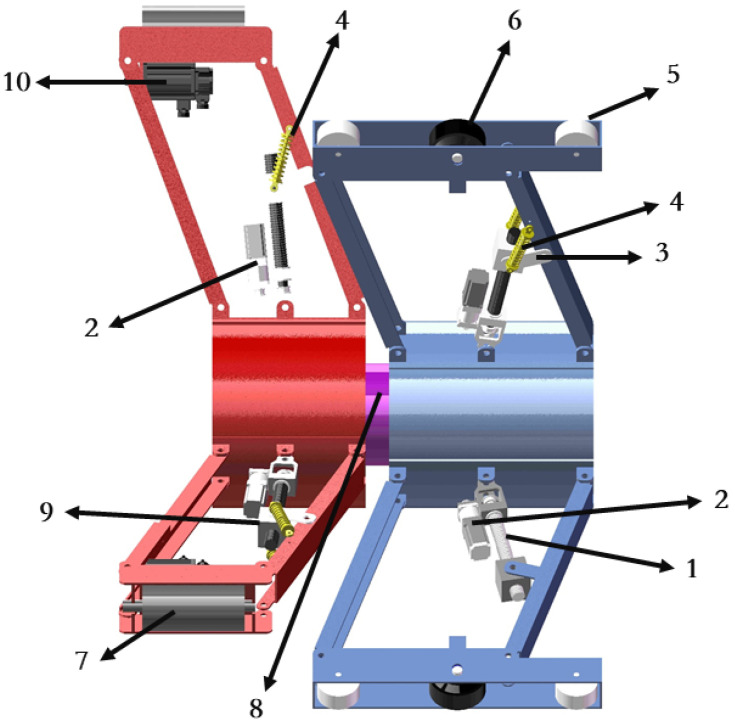
Pipeline inner wall polishing robot. 1. Lead screw; 2. Stepper motor with gearbox; 3. Hinge; 4. Shock-absorbing spring; 5. Support wheel; 6. Hub motor; 7. Roller steel brush; 8. Slewing bearing; 9. Lead screw nut; 10. Servo motor.

The pipeline robot designed in this paper is intended for grinding the inner walls of prestressed concrete cylinder pipes (PCCP) with diameters of 1600–2400 mm. It needs to enter the pipeline through an 800 mm diameter exhaust valve, and its structure is simple, making it easy to assemble and disassemble. During the traveling grinding operation, it must adapt to variations in the pipe’s inner wall, be able to pass through protrusions or local deposits inside the pipeline, and navigate through bends. The pipeline robot designed according to these operational requirements is shown in Figure 1, where the blue part represents the walking support section and the red part represents the rotating grinding section, connected through a slewing bearing.

### 2.2. Design and Working Principle of the Support Walking Mechanism

**Figure 2:**
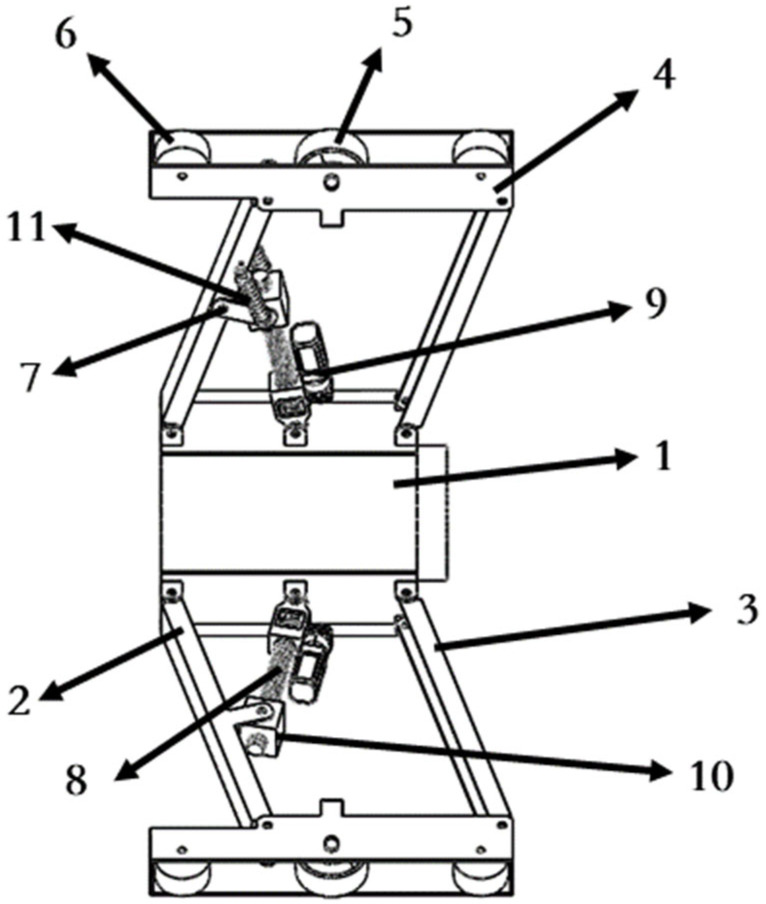
Diagram of the walking support structure. 1. Main part of the walking mechanism; 2. Rear supporting leg; 3. Front supporting leg; 4. Wheel frame; 5. Drive wheel; 6. Support wheel; 7. Hinge; 8. Lead screw; 9. Stepper motor with reducer; 10. Lead screw nut; 11. Shock-absorbing spring.

**Figure 3:**
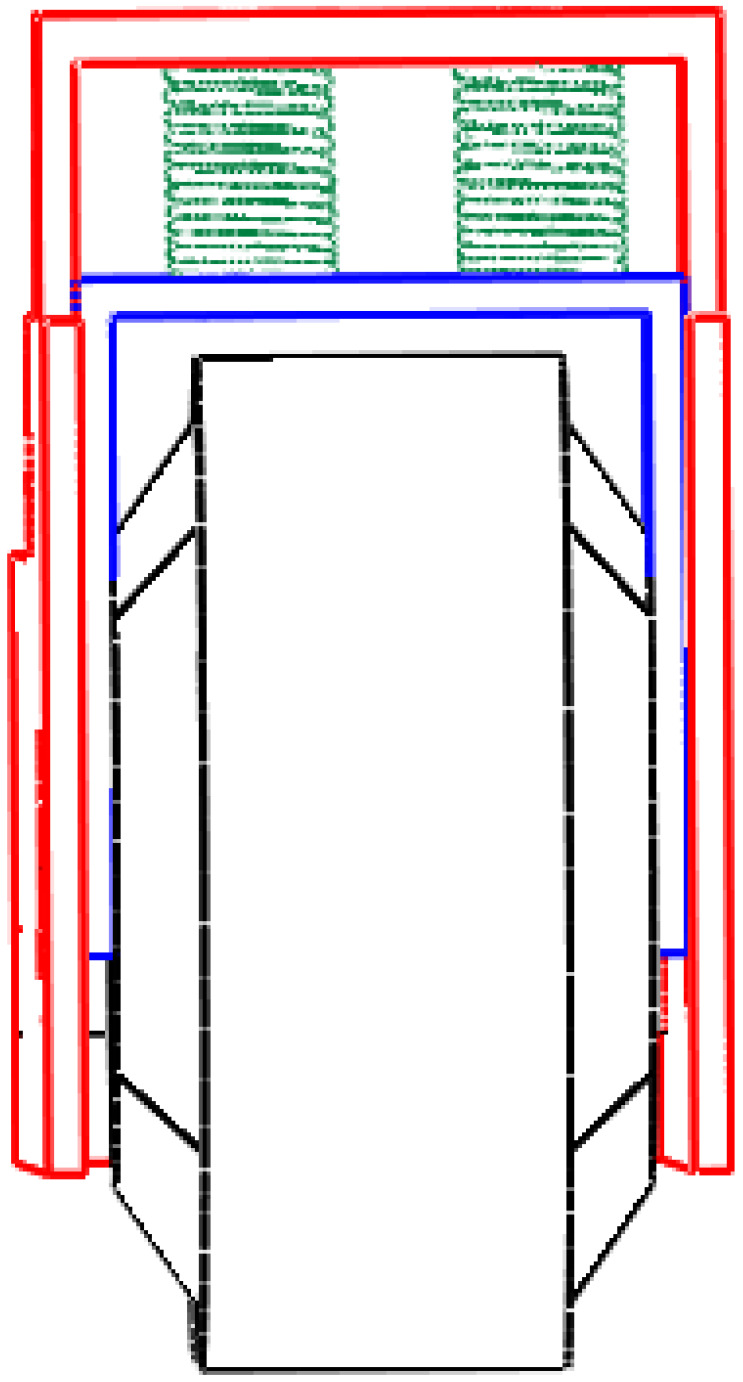
Schematic diagram of the drive wheel structure.

As shown in Figure 2, the support and walking mechanism of the pipeline robot consists of four walking support wheel assemblies, which are distributed around the circumference at 90° intervals. The wheel frame, the front and rear support legs, and the walking section of the body form a four-bar linkage. Each support wheel leg is supported by a lead screw, with a stepper motor transmitting power through a reducer and driving the lead screw via a chain, enabling the support wheel legs to extend and retract. The upper two-wheel leg supports differ from the lower two. The upper lead screw is flexibly connected to the rear support leg through a hinge and a shock-absorbing spring, allowing the walking mechanism to dynamically adapt to changes in the inner surface of the pipe; the lower lead screw is rigidly connected to the rear support leg. Each wheel assembly consists of three wheels, with the middle one being the drive wheel. The installation structure of the drive wheel is shown schematically in Figure 3, with the front and rear wheels serving as support wheels. The drive wheel is connected to the wheel frame via two compression springs, which compress to overcome raised obstacles while maintaining contact with the pipe, ensuring that power is not lost.

### 2.3. Design and Working Principle of the Support Walking Mechanism

**Figure 4:**
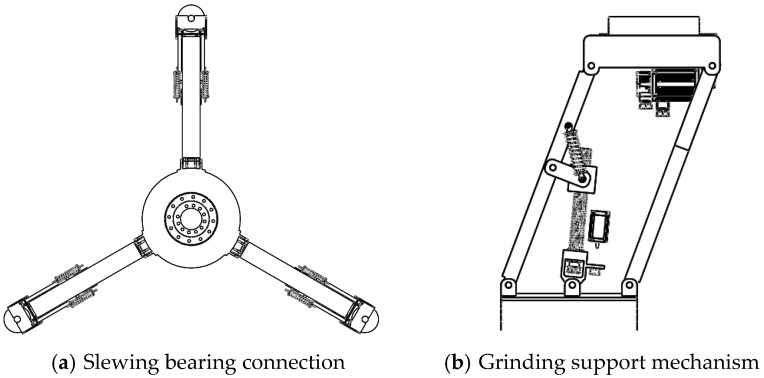
Polishing the module structure diagram.

The schematic diagram of the grinding module structure is shown in Figure 4. The main body of the grinding module is connected to the slewing bearing, and the grinding mechanism consists of three grinding sets arranged circumferentially at 120° intervals. The steel brush holder, front and rear support legs, and the main body form a four-bar linkage. The lead screw nut is flexibly connected to the rear support leg through a hinge and a spring damper. During the grinding operation, the main body drives the three grinding sets to rotate, while the three-roller steel brushes rotate at high speed, performing the grinding of the inner wall of the pipeline.

### 2.4. Chapter Summary

This section presents the overall configuration of a novel grinding robot for the inner wall of PCCP pipes. The design consists of two main modules: a walking/support module and a rotary grinding module, and the working principle of the mechanism is also described.

## 3. Analysis of Motion Characteristics of Inner Wall Grinding Robot for PCCP Pipes

### 3.1. Dynamic Coupling Model for Synergistic Force Bearing of Four Supporting Mechanisms in a Pipeline Robot

**Figure 5:**
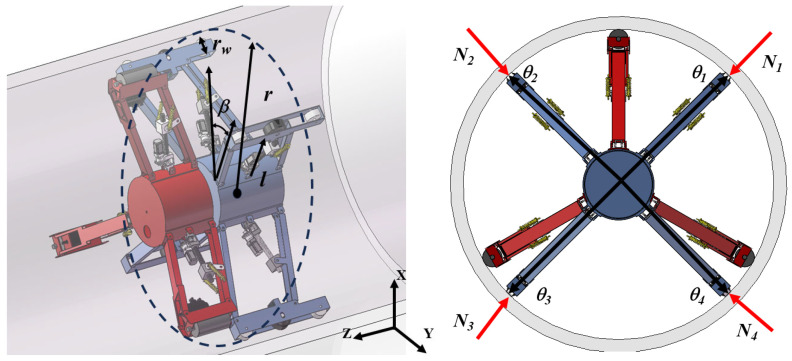
Schematic diagram of the dynamic model.

As shown in Figure 5, Taking the center of the cylindrical main body as the origin, a cylindrical coordinate system ***r***, ***θ***, ***z*** is established, where the z-axis is along the pipeline axis, the θ-axis is circumferential (the circumferential angles of the four supporting mechanisms are $\theta_{1}=0^{\circ },\theta_{2}=90^{\circ },\theta_{3}=180^{\circ },\theta_{4}=270^{\circ }$ in sequence), and the r-axis points radially to the inner wall of the pipeline. The model integrates three parts: synergistic constraint of radial supporting forces, coupled transmission of axial driving forces, and dynamic balance of the overall attitude, which are detailed as follows:

1. Synergistic Constraint Equation of Radial Supporting Forces (Core of Centering Constraint).

The radial contact-reaction forces $N_{i}$ (i=1,2,3,4) of the four supporting mechanisms must satisfy the condition that the resultant radial force is zero to ensure no radial deviation of the robot inside the pipeline. The synergistic constraint equations are:

(1) $$\left\{\begin{array}{c} \sum_{i=1}^{4}N_{i}\cos\theta_{i}=0\\ \sum_{i=1}^{4}N_{i}\sin\theta_{i}=0\;, \end{array}\right.$$

Substituting the circumferential angles $\theta_{i}$, the specific form is obtained as:

(2) $$\left\{\begin{array}{l} N_{1}-N_{3}=0\\ N_{2}-N_{4}=0\;, \end{array}\right.$$

The mechanical relationship between the radial contact reaction force $N_{i}$ of a single four-bar mechanism and the supporting drive pair torque $T_{si}$ is:

(3) $$N_{i}=\frac{T_{si}\cdot \eta_{i}}{l_{i}\cdot \sin\beta_{i}},$$

where:

$\eta_{i}$: Force transmission efficiency of the four-bar mechanism (considering hinge friction of rods, 0.85∼0.95);

$l_{i}$: Length of the force arm from the drive pair of the four-bar mechanism to the wheel contact point;

$\beta_{i}$: Angle between the drive rod of the four-bar mechanism and the radial direction;

2. Coupled Transmission Equation of Axial Driving Forces

The axial driving force $F_{di}$ of the driving wheel is provided by the motor torque $T_{di}$ and is limited by the maximum static friction force determined by the radial contact reaction force $N_{i}$. The driving forces of the four mechanisms synergistically satisfy:

(4) $$\sum_{i=1}^{4}F_{di}=\sum_{i=1}^{4}\frac{T_{di}\cdot \eta_{d}}{r_{w}}F_{di}\leq \mu_{s}N_{i},$$

where:

$\eta_{d}$: Transmission efficiency of the drive system (including reducer and wheel train friction, 0.8∼0.9);

$r_{w}$: Effective radius of the driving wheel;

$\mu_{s}$: Static friction coefficient between the wheel and the pipe wall (to be measured experimentally, 0.15∼0.3 for metal-metal contact surfaces);

Combined with the overall axial translational dynamic equation, the coupled dynamic balance equation is obtained as:

(5) $$\sum_{i=1}^{4}\frac{T_{di}\cdot \eta_{di}}{r_{w}}-\sum_{i=1}^{4}f_{i}N_{i}-F_{fl}=m\ddot{z},$$

where $f_{r}$ is the rolling friction coefficient, $F_{fd}$ is the axial viscous resistance, m is the total mass of the robot, and $\ddot{z}$ is the axial acceleration.

3. Coupled Dynamic Equation of the Overall Attitude Around the Axial Direction

The speed difference between the four driving wheels will generate a disturbance torque around the z-axis, leading to circumferential rotation of the robot. The attitude dynamic coupling equation is

(6) $$\sum_{i=1}^{4}\left(T_{di}-T_{fri}\right)\cos\gamma_{i}-F_{fl}h_{g}=J_{z}\ddot{\omega },$$

where:

$T_{fri}$: Friction resistance torque of a single wheel around its axle;

$\gamma_{i}$: Angle between the direction of the driving torque and the circumferential direction ($\gamma_{i}=0^{\circ }$ for symmetrical distribution);

$J_{z}$: Moment of inertia of the entire robot around the z-axis (superimposed by the moment of inertia of the cylindrical main body $J_{z0}$ and the moments of inertia of the four supporting mechanisms $J_{zi}$, $J_{z}=J_{z0}+\sum_{i=1}^{4}J_{zi}$);

$\ddot{\omega }$: Angular acceleration around the z-axis.

### 3.2. Analysis of Walking and Supporting Motion

**Figure 6:**
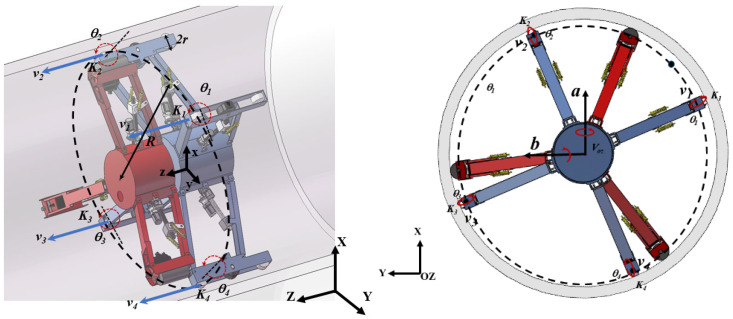
Kinematic model of pipeline robot. Parameter description: ***V*_1_**–***V*_4_**—Linear speed of the driving wheel, ***K*_1_**–***K*_4_**—Contact point between the driving wheel and the inner pipe wall, ***θ*_1_**–***θ*_4_**—Angular velocity of the driving wheel, ***R***—Pipe radius, ***a***—Unit vector in the x-direction, ***b***—Unit vector in the y-direction.

The kinematic model of the pipeline robot was established as illustrated in Figure 6. Given that the robot’s supporting wheel assemblies are configured in four sets circumferentially distributed at 90° intervals, with each set driven by an independent motor, its instantaneous motion inside a straight pipe can be decomposed into two components: translational motion along the pipe axis and rotational motion about two axes perpendicular to the direction of translation. In this model, OXYZ denotes the global reference frame, while oxyz represents the local coordinate system anchored at the robot’s center. The parameters a, b, and c are the unit vectors of the local coordinate system, respectively; moreover, the *x*-axis of the local coordinate system remains vertically upward regardless of the robot’s attitude angle.

First, the following assumptions are made: each wheel assembly remains in constant contact with the inner wall of the pipe without horizontal slippage or rotation about the *z*-axis and only moves along the *z*-axis direction. Let ***θ*_1_**, ***θ*****_2_**, ***θ*****_3_**, and ***θ*_4_** denote the angular velocities of the four-wheel assemblies, respectively; ***r*** is the radius of the driving wheel; ***R*** is the radius of the robot itself; and **β** is the attitude angle of the robot inside the pipe. Based on the above definitions, the linear velocities ***V*****_1_**, ***V*****_2_**, ***V*_3_**, and ***V*_4_** of the four-wheel assemblies can be expressed as follows:

(7) $$\begin{array}{l} V_{1}=r\theta_{1}\\ V_{2}=r\theta_{2}\\ V_{3}=r\theta_{3}\\ V_{4}=r\theta_{4} \end{array},$$

**Figure 7:**
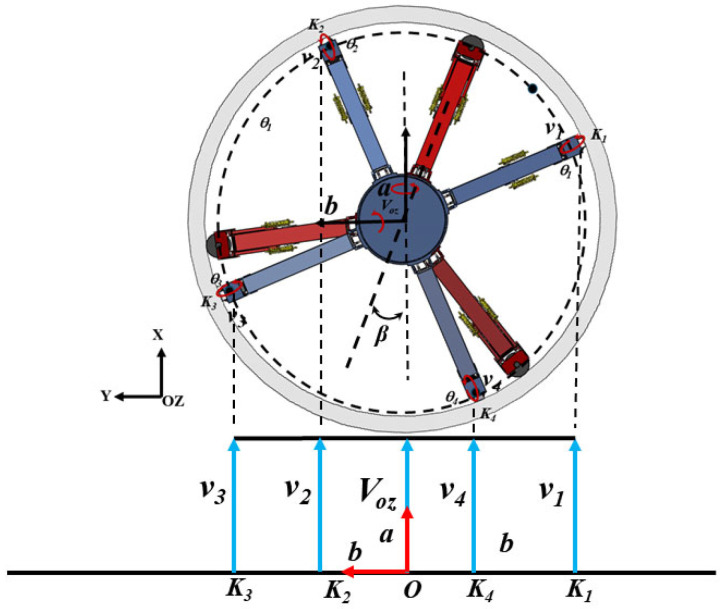
Velocity profile. Parameter Description: ***V_oz_***—Velocity of the local coordinate system origin along the Z-direction, ***β***—Pose angle of the pipeline robot, ***ω_oz_***—Angular velocity of the pipeline robot about the local coordinate system, ***ω_a_***—Angular velocity of the pipeline robot about the *x*-axis of the local coordinate system, ***ω_b_***—Angular velocity of the pipeline robot about the *y*-axis of the local coordinate system.

A locomotion model of the pipeline robot was established, with the mapping of its cross-sectional view and velocity profile diagram illustrated as shown in Figure 7. ***V_oz_*** denotes the linear velocity at the robot’s center, while ***ω_a_*** and ***ω_b_*** represent the angular velocities of the robot rotating around the *x*-axis and *y*-axis of the local coordinate system, respectively. To derive the kinematic relationship between the angular velocities of the wheel assemblies (***θ*_1_**, ***θ*_2_**, ***θ*_3_**, and ***θ*_4_**) and the motion velocities of the pipeline robot (***ω_a_***, ***ω_b_***, and ***V_oz_***), an analysis was conducted on four different velocity states.

(1) *V*_1_ = *V*_2_ = *V*_3_ = *V*_4_: The linear velocities of the four-wheel assemblies of the pipeline robot are equal, and the robot moves forward steadily. In this case, the rotational velocities *ω_a_* and *ω_b_* are zero, meaning the robot performs no deflecting motion inside the pipe. The linear velocity *V_oz_* at the centroid O of the robot can be expressed as follows:

(8) $$V_{OZ}=V_{1}=V_{2}=V_{3}=V_{4}=\theta r\;\begin{array}{l} \omega_{x}=0\\ \omega_{y}=0 \end{array},$$

**Figure 8:**
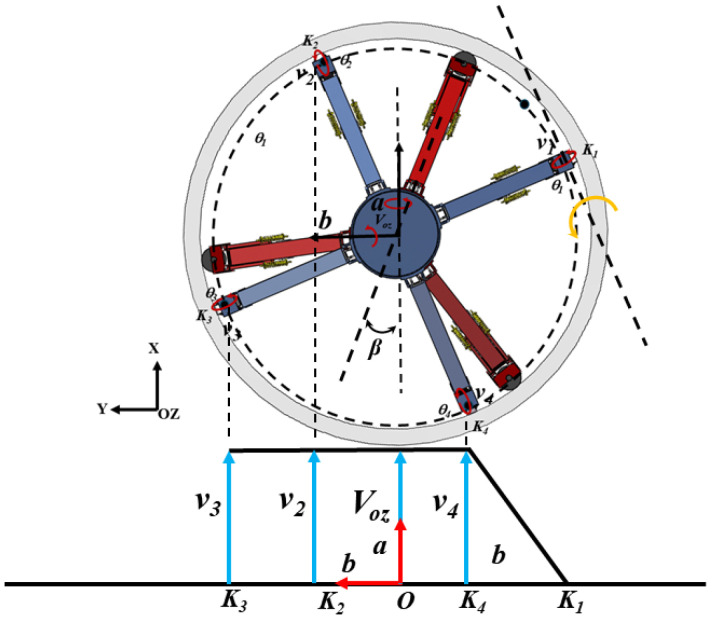
Velocity Profile.

(2) *V*_1_ = 0, *V*_2_ = *V*_3_ = *V*_4_ ≠ 0: As shown in Figure 8, Three-wheel assemblies of the robot have linear velocities, while point K1 remains stationary. Linear velocities *V_2_*, *V_3_*, and *V_4_* are generated at points *K*_2_, *K*_3_, and *K*_4_, respectively. If the velocities at these three points are equal (*V_2_* = *V*_3_ = *V*_4_ ≠ 0), the linear velocity *V_oz_* at the robot’s center O can be derived as *V_oz_* = *V_2_* through geometric analysis. Therefore, the rotational velocity *ω_oz_* of the robot’s center O about point *K*_1_ can be expressed as follows:

(9) $$\omega_{\mathrm{oz}}=\frac{V_{2}}{R},$$

The angular velocity vector ωoz of the local coordinate system on the robot can be expressed as follows:

(10) $$\omega_{\mathrm{oz}}=\frac{r\theta_{2}}{R}\left[\cos(45{}^{\circ}-\beta )\vec{b}-\sin(45{}^{\circ}-\beta )\vec{a}\right]=\cos(45{}^{\circ}-\beta )\frac{\theta_{2}r}{R}\vec{b}-\sin(45{}^{\circ}-\beta )\frac{\theta_{2}r}{R}\vec{a},$$

(11) $$\begin{array}{l} \omega_{x}=\cos(45{}^{\circ}-\beta )\frac{\theta_{2}r}{R}\\ \omega_{y}=-\sin(45{}^{\circ}-\beta )\frac{\theta_{2}r}{R} \end{array},$$

In this case, the robot rotates about the straight line passing through points K1 and K2. The geometric expression for the linear velocity ***V_oz_*** of the robot’s center is given by:

(12) $$V_{oz}=\frac{\sqrt{2}}{2}V_{3}=\frac{\sqrt{2}}{2}r\theta_{3},$$

**Figure 9:**
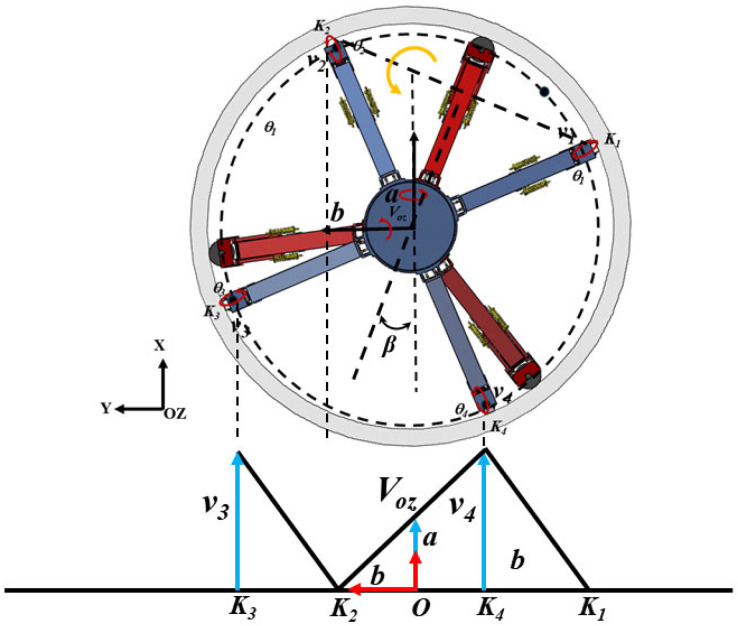
Velocity Profile.

(3) *V*_1_ = *V*_2_ = *0*, *V*_3_ = *V*_4_ ≠ 0: As shown in Figure 9, Only two-wheel assemblies of the robot have linear velocities, while points *K*_1_ and *K*_2_ remain stationary. Linear velocities *V*_3_ and *V*_4_ are generated at points *K*_3_ and, only one wheel assembly of the robot has a *K*_4_, respectively; if the velocities at these two points are equal (*V*_3_ = *V*_4_ ≠ *0*), the rotational velocity *ω*_34_ is induced by the linear velocities *V*_3_ and *V*_4_. Through geometric analysis, the linear velocity *Voz* at the robot’s center O can be derived as *V_oz_* = $\left(\sqrt{2}/2\right)$*V*_3_. The rotational velocity vector *ωoz* of the local coordinate system on the robot can be expressed as follows:

(13) $$\omega_{\mathrm{oz}}=\frac{r\theta_{3}}{\sqrt{2}R}\left[-\sin\beta \vec{b}-\cos\beta \vec{a}\right]=-\cos\beta \frac{\theta_{3}r}{\sqrt{2}R}\vec{a}-\sin\beta \frac{\theta_{3}r}{\sqrt{2}R}\vec{b},$$

(14) $$\begin{array}{l} \omega_{x}=-\sin\beta \frac{\theta_{3}r}{\sqrt{2}R}\\ \omega_{y}=-\cos\beta \frac{\theta_{3}r}{\sqrt{2}R} \end{array},$$

In this case, the robot rotates about the straight line passing through points ***K*_1_** and ***K*_2_**. The geometric expression for the linear velocity ***V_oz_*** at the robot’s center is given by:

(15) $$V_{oz}=\frac{\sqrt{2}}{2}V_{3}=\frac{\sqrt{2}}{2}r\theta_{3},$$

(4) ***V*_1_** =***V*****_2_** =***V*****_3_** = **0**, ***V*****_4_** ≠ **0**: In this case, only one wheel assembly of the robot has a linear velocity. Points ***K*_1_**, ***K*_2_**, and ***K*_3_** remain stationary while a linear velocity ***V*_4_** is generated at point ***K*_4_**. Through geometric analysis, it is derived that the linear velocity ***V_oz_*** at the robot’s center O is zero. The rotational velocity vector ***ω_oz_*** of the local coordinate system on the robot can be expressed as follows:

(16) $$\omega_{\mathrm{oz}}=\frac{r\theta_{3}}{R}\left[-\sin(45{}^{\circ}-\beta )\vec{b}-\cos(45{}^{\circ}-\beta )\vec{a}\right]=-\cos(45{}^{\circ}-\beta )\frac{\theta_{3}r}{R}\vec{a}+\sin(45{}^{\circ}-\beta )\frac{\theta_{3}r}{R}\vec{b},$$

(17) $$\begin{array}{l} \omega_{x}=\sin(45{}^{\circ}-\beta )\frac{\theta_{3}r}{R}\\ \omega_{y}=-\cos(45{}^{\circ}-\beta )\frac{\theta_{3}r}{R} \end{array},$$

By systematically analyzing the motion characteristics of the pipeline robot under four operating conditions, a theoretical model correlating the input velocities of the supporting wheel assemblies with the overall locomotion velocity of the robot was established. Based on the derived results of this model, the influence law of velocity differences among individual wheel assemblies on the robot’s locomotion attitude inside the pipeline can be clearly and explicitly revealed. The purpose of constructing this model is to provide reliable theoretical support for the precise regulation of the robot’s locomotion attitude, ensure that the robot travels stably at the desired velocity in complex pipeline environments, and lay a solid foundation for the smooth implementation of subsequent grinding operations.

**Figure 10:**
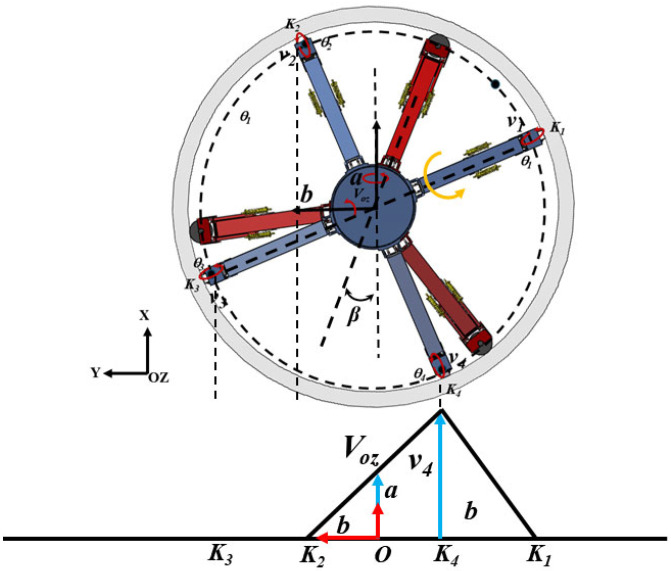
Velocity profile.

The reliability and stability of the diameter-varying motion are crucial to the overall operation of the pipeline robot. To systematically investigate the mechanical characteristics of this transmission system, a force analysis was carried out on the supporting diameter-varying mechanism, as illustrated in Figure 10. The pipeline robot makes contact with the inner wall of the pipe via four sets of supports and bears the corresponding reaction forces ***F*****_1_**, ***F*****_2_**, ***F*****_3_**, and ***F*_4_** from the inner wall. Meanwhile, the entire robot is subjected to gravity, and, thus, the supporting lead screws on the lower side are required to provide an axial force ***F_z_***.

A plane Cartesian coordinate system is established with point A as the origin, where the positive *y*-axis is directed vertically downward, and the positive *x*-axis is directed horizontally to the left. Given the coordinates of point B as (***L_ab_cosθ***, ***L_ab_sinθ***), let the coordinates of point C be (***x_c_***, ***y_c_***). Then, the vector AB can be expressed as (***L_ab_cosθ***, ***L_ab_sinθ***), and the vector BC as (***x_c_*** − ***L_ab_cosθ***, ***y_c_*** − ***L_ab_sinθ***). According to the geometric constraint conditions, vectors AB and BC are perpendicular to each other, and the length of segment BC is ***L_bc._***

According to the static equilibrium equations,

(18) $$\left\{\begin{array}{l} {\left(x_{c}-L_{ab}\cos\theta \right)}^{2}+{\left(y_{c}-L_{ab}\sin\theta \right)}^{2}=L_{bc}^{2}\\ L_{ab}\cos\theta \cdot (x_{c}-L_{ab}\cos\theta )+L_{ab}\sin\theta \cdot (y_{c}-L_{ab}\sin\theta )=0 \end{array}\right.\;,$$

(19) $$\left\{\begin{array}{l} x_{c}=L_{ab}\cos\theta +L_{bc}\sin\theta \\ y_{c}=L_{ab}\sin\theta -L_{bc}\cos\theta \end{array}\right.\;,$$

The Cartesian coordinates of point C are (***L_ab_cosθ*** + ***L_bc_sinθ***, ***L_ab_sinθ*** − ***L_bc_cosθ***).

The formula for the axial force transmitted by the lead screw is as follows:

(20) $$F_{z}=\frac{2\pi \eta T}{p_{h}},$$

Parameter Description

η—Transmission efficiency of the lead screw

$p_{h}$—Lead of the lead screw

T—Output torque of the motor

Conversion Formula for Motor Power, Rotational Speed, and Torque:

(21) $$T=9550\frac{P}{n},$$

Parameter Description

***P***—Motor power

***n***—Motor rotational speed

***T***—Output torque

**Figure 11:**
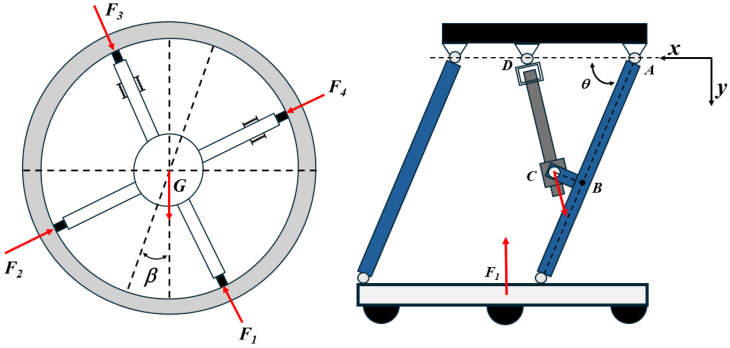
Force Analysis Diagram. Parameter Description: ***F*_1_**–***F***_4_—Reaction forces exerted by the inner pipe wall on the four support-wheel sets, ***β***—Pose angle of the pipeline robot, ***θ***—Inclination angle of support rod AB with respect to the horizontal, ***G***—Body weight.

Based on the above formula derivation and the geometric relationships shown in Figure 11, the torque relationship of the lead screw (Equation (20)) and the motor selection power relationship (Equation (21)) can be deduced.

(22) $$T=\frac{p_{h}\left(\frac{G}{\cos(45{}^{\circ}\;-\;\beta )}\;+F_{3}\right)}{2\pi \eta \sin\left[\arccos\left(\frac{L_{ad}\;-\;L_{ab}\cos\theta \;-\;L_{bc}\sin\theta }{\sqrt{{\left(L_{ad}\;-\;L_{ab}\cos\theta \;-\;L_{bc}\sin\theta \right)}^{2}\;+\;{\left(L_{bc}\cos\theta \;-\;L_{ab}\sin\theta \right)}^{2}}}\right)\right]},$$

(23) $$P=\frac{np_{h}\left(\frac{G}{\cos(45{}^{\circ}\;-\;\beta )}+F_{3}\right)}{19100\pi \eta \sin\left[\arccos\left(\frac{L_{ad}\;-\;L_{ab}\cos\theta \;-\;L_{bc}\sin\theta }{\sqrt{{\left(L_{ad}\;-\;L_{ab}\cos\theta \;-\;L_{bc}\sin\theta \right)}^{2}\;+\;{\left(L_{bc}\cos\theta \;-\;L_{ab}\sin\theta \right)}^{2}}}\right)\right]},$$

As the core transmission and load-bearing component of the supporting diameter-varying device, the lead screw-nut mechanism undertakes the key function of converting the rotational motion of the motor into linear motion. The output torque of the motor is transmitted through the lead screw and converted into an axial driving force, thereby achieving the telescopic diameter variation of the supporting arm. In this section, the axial load to be borne by the lead screw is determined through force analysis, and then the torque required to drive the rotation of the lead screw is derived from the axial load. Finally, the motor power meeting the operating condition requirements is obtained through inverse calculation, which provides key dynamic parameter support for the structural design of the supporting diameter-varying device and the subsequent selection of core components.

### 3.3. Analysis of Traction Performance

When a pipeline robot operates inside a pipeline, the combined effects of its own weight and tension generate support forces from the inner wall of the pipeline on its wheel sets. The magnitude of these support forces directly affects the traction force. Different posture angles result in varying support forces on the four-wheel sets within the pipeline. Therefore, the traction output of the pipeline robot is influenced by its own operational posture angle [18,19,20,21].

**Figure 12:**
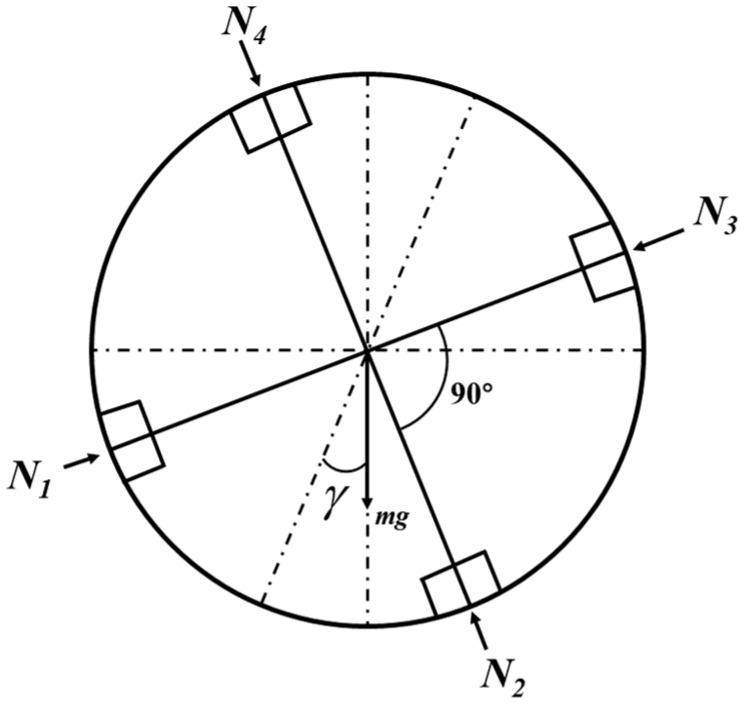
Pipe Robot Stress Diagram. Parameter Description, ***N*_1_**–***N*_4_**—Reaction force from the inner pipe wall on the pipeline robot, ***γ***—Attitude angle of the pipeline robot, ***Mg***—Weight of the pipeline robot.

In order to study the optimal working posture angle of the pipeline robot, the forces on the robot are analyzed. Its force conditions inside the pipeline are shown in Figure 12. Starting from the lower-left wheel set and moving counterclockwise are wheel sets 1 to 4. The support forces from the pipe wall on each wheel set are ***N*_1_**, ***N*****_2_**, ***N*****_3_**, and ***N*_4_**. The gravitational force acts vertically downward. γ is the posture angle of the pipeline robot, and the angle between two adjacent support wheel sets is. The sum of the support forces of the four-wheel sets ***N*_1_**, ***N*****_2_**, ***N*****_3_**, and ***N*_4_** of the pipeline robot can be expressed as:

(24) $$\sum_{i=1}^{4}N_{i}=N_{1}+N_{2}+N_{3}+N_{4},$$

List the force equilibrium equations according to Figure 12:

(25) $$\left\{\begin{array}{l} N_{1}\cos(45^{{}^{\circ}}+\gamma )+N_{2}\cos(45^{{}^{\circ}}-\gamma )-mg-N_{4}\cos(45^{{}^{\circ}}-\gamma )-N_{3}\cos(45^{{}^{\circ}}+\gamma )=0\\ N_{1}\sin(45^{{}^{\circ}}+\gamma )+N_{4}\sin(45^{{}^{\circ}}-\gamma )-N_{2}\sin(45^{{}^{\circ}}-\gamma )-N_{3}\sin(45^{{}^{\circ}}+\gamma )=0 \end{array}\right.\;,$$

Under the combined action of gravity and tension force, the pipeline robot forms a statically indeterminate structure as a whole. The two wheel sets on the lower side serve as the primary supporting components, while the two wheels on the upper side only function to prevent the robot from rolling over. To facilitate the analysis of the relationship among the attitude angle, supporting force, and traction force, we assume the upper supporting forces N_3_ = 0 and N_4_ = 0. (This assumption corresponds to the limiting case where the preload in the two upper supporting structures is zero.) Substituting these conditions into Equation (25) yields:

(26) $$\left\{\begin{array}{l} N_{1}\cos(45^{{}^{\circ}}+\gamma )+N_{2}\cos(45^{{}^{\circ}}-\gamma )-mg=0\\ N_{1}\sin(45^{{}^{\circ}}+\gamma )-N_{2}\sin(45^{{}^{\circ}}-\gamma )=0 \end{array}\right.\;,$$

By simplifying, we get:

(27) $$N_{1}+N_{2}=\sqrt{2}mg\cos\gamma \;,$$

From Equation (27), it can be seen that when γ=0, the sum of the supporting forces on the four-wheel sets is maximized. At this point, the traction force of the pipeline robot also reaches its maximum. Therefore, γ=0 is the optimal attitude angle for the pipeline robot during operation.

The traction capability of a pipeline robot directly affects its load capacity and is a key indicator of its ability to navigate pipelines. The resistances encountered by the pipeline robot designed in this study include: wheelset frictional resistance, drag resistance, and grinding resistance. The drag resistance and grinding resistance are much smaller than the wheelset frictional resistance; therefore, this study focuses primarily on analyzing the frictional resistance of the pipeline robot. There are two situations for the frictional resistance experienced by the pipeline robot designed in this study: one is the friction generated by the support wheelset against the inner wall of the pipeline due to the robot’s own weight, and the other is the friction caused by the clamping force generated from the additional tension provided by the robot’s support components. Considering only the effect of gravity, the forces acting on the pipeline robot are:

(28) $$\left\{\begin{array}{c} N_{1}\cos(\gamma +45^{{}^{\circ}})+N_{2}\cos(45^{{}^{\circ}}-\gamma )=mg\\ N_{1}\sin(\gamma +45^{{}^{\circ}})-N_{2}\sin(45^{{}^{\circ}}-\gamma )=0 \end{array}\right.,$$

Solve for ***N*_1_** and ***N*_2_** to obtain

(29) $$\left\{\begin{array}{c} N_{1}=mg\cos(45^{\circ }+\gamma )\\ N_{2}=mg\cos(45^{\circ }-\gamma ) \end{array}\right.,$$

When only gravity is acting, the traction force can only be provided by Wheel 1 and Wheel 2. The spring preload force for driving Wheel 1 and Wheel 2 is:

(30) $$\left\{\begin{array}{l} F_{s1}=N_{1}=mg\cos(45^{{}^{\circ}}+\gamma )\\ F_{s2}=N_{2}=mg\sin(45^{{}^{\circ}}-\gamma ) \end{array}\right.,$$

According to the previous analysis, it can be seen that under the condition of tension, the entire machine structure is a statically indeterminate structure, and the traction is provided collectively by the four wheelsets. Assuming the tension of supporting wheelsets 3 and 4 is, the force equilibrium equations are listed according to Figure 12:

(31) $$\left\{\begin{array}{l} N_{1}\cos(45^{{}^{\circ}}+\gamma )+N_{2}\cos(45^{{}^{\circ}}-\gamma )-mg-F_{4}\cos(45^{{}^{\circ}}-\gamma )-F_{3}\cos(45^{{}^{\circ}}+\gamma )=0\\ N_{1}\sin(45^{{}^{\circ}}+\gamma )+F_{4}\sin(45^{{}^{\circ}}-\gamma )-N_{2}\sin(45^{{}^{\circ}}-\gamma )-F_{3}\sin(45^{{}^{\circ}}+\gamma )=0 \end{array}\right.\;,$$

Solve for ***N*_1_** and ***N*_2_**:

(32) $$\left\{\begin{array}{l} N_{1}=mg\sin(45^{{}^{\circ}}-\gamma )+F_{3}\\ N_{2}=mg\sin(45^{{}^{\circ}}+\gamma )+F4 \end{array}\right.,$$

The minimum traction force (the maximum static friction force) depends on the magnitude of the tension force from the two upper supporting wheel sets. The spring tension forces of the four driving wheels are:

(33) $$\left\{\begin{array}{l} F_{s1}=N1=mg\cos(45^{{}^{\circ}}-\gamma )+F_{3}\\ F_{s2}=N2=mg\sin(45^{{}^{\circ}}+\gamma )+F_{4}\\ F_{s3}=F_{3}\\ F_{s4}=F_{4} \end{array}\right.,$$

Parameter Description

***Fs*_1_**—Spring preload forces of the four driving wheels.

### 3.4. Analysis of Towing Cable Resistance

The pipeline robot designed in this paper has higher volume and power requirements than traditional pipeline robots. The battery-powered self-supply mode struggles to meet the demand for continuous operation and is prone to shutdown failures inside the pipeline, which in turn severely limits the robot’s working duration and operational stability. Meanwhile, the complex internal environment of the pipeline and severe signal attenuation lead to poor stability of wireless communication methods, failing to guarantee the reliability of data transmission. Based on the above analysis and considering the characteristic of high-power consumption of components such as motors equipped on the robot, a wired drive scheme is adopted in practice, where energy transmission and communication with the upper computer are realized synchronously through cables. However, this scheme has obvious drawbacks: affected by the friction between the cable and the pipe wall and the gradual accumulation of the cable’s own weight, the towing resistance of the cable increases continuously with the extension of the operation distance. In consideration of pipeline differences, the towing resistance of the cable under straight pipe and curved pipe conditions is analyzed in this section.

When the pipeline robot travels in a straight pipe, the cable moves synchronously with the robot and trails behind the body. Combining with the actual operation scenario, the cable can be divided into two segments: one is the suspended segment connected to the robot body, and the other is the contact segment closely attached to the inner wall of the pipe. To analyze the cable towing resistance, this paper assumes that the robot moves at a constant speed, selects an infinitesimal cable segment as the research object, establishes a force equilibrium equation for this infinitesimal segment, and derives the towing resistance generated by the cable segment closely attached to the inner pipe wall through integral operation.

**Figure 13:**
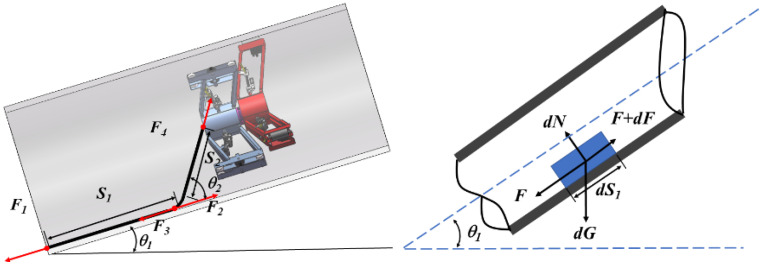
Force Analysis Diagram. Parameter description: ***θ*****_1_**—Inclination angle of straight pipe, ***θ*****_2_**—Angle between suspended cable segment S_2_ and pipe axis, ***S*****_1_**, ***S*_2_**—Cable length, ***F*_1_**, ***F*_2_**—Tensions at the ends of cable segment S_1_ in contact with the pipe wall, ***F*****_3_**, ***F*_4_**—Tensions at the ends of cable segment ***S2*** in contact with the pipe wall, ***F***—Tension at lower end of segment, ***F*** + ***dF***—Tension at upper end of segment, ***dG***—Length of infinitesimal cable segment, ***μ***—Friction coefficient between cable and inner pipe wall, ***r***—Cable radius, ***Js***—Moment of inertia of the suspended segment, $\ddot{\theta }$—Angular acceleration.

The cables in the straight section of the pipeline experience stress as shown in Figure 13. The slope angle of the straight pipe is ***θ*_1_**, and the angle between the suspended cable segment ***S*_2_** and the pipe is ***θ*_2_**. ***F*_1_** and ***F*_2_** are the tensile forces acting on the two ends of the cable segment attached to the pipe, while ***F*_3_** and ***F*_4_** are the tensile forces acting on the two ends of the suspended cable segment. An infinitesimal segment of the cable ***S*_1_** is selected for analysis, as shown in the diagram. The length of the infinitesimal segment is ***dS*_1_**, the supporting force exerted by the pipe wall on the infinitesimal cable segment is ***dN***, the gravity of the infinitesimal segment is ***dG***, the friction coefficient between the cable and the inner pipe wall is **μ**, and the tensile forces acting on its two ends are ***F*** and ***F*** + ***dF***.

According to the force equilibrium conditions, the following equations can be obtained:

(34) $$\left\{\begin{array}{l} F+\mu dN+dG\sin\theta_{1}=F+dF\\ dN=dG\cos\theta_{1}\\ dG=\rho \pi r^{2}gdS_{1} \end{array}\right.\;,$$

From the above system of equations, it can be concluded that:

(35) $$dF=\rho \pi r^{2}g\left(\mu \cos\theta_{1}+\sin\theta_{1}\right)dS_{1},$$

Integrating both sides of the equation yields:

(36) $$F=\rho \pi r^{2}g\left(\mu \cos\theta_{1}+\sin\theta_{1}\right)S_{1},$$

Therefore:

(37) $$F_{2}=F_{1}+\rho \pi r^{2}g\left(\mu \cos\theta_{1}+\sin\theta_{1}\right)S_{1},$$

With the friction coefficient, cable parameters, and pipe slope angle determined, $\rho \pi r^{2}g\left(\mu \cos\theta_{1}+\sin\theta_{1}\right)$ is a constant overall, and the towing resistance of cable segment S1 is only related to the cable length and the tensile force acting on the cable.

Owing to the distance between the cable attachment point and the pipe wall, the suspended cable segment is unavoidable. There exists an arc transition section between the suspended segment and the wall-attached segment, which is negligible relative to the total length of the pipeline. Taking an instantaneous moment when the robot moves at a constant speed, the motion of the suspended cable segment can be decomposed into two instantaneous motions: translational motion following the robot and rotational motion around the attachment point. Let the moment of inertia of the suspended cable segment be ***J_s_***, and its angular acceleration be $\ddot{\theta }$. By uniformly taking moments about the centroid, the following equation can be obtained:

(38) $$\left\{\begin{array}{l} F_{4}\cos\left(\theta_{1}+\theta_{2}\right)=F_{3}\cos\theta_{1}\\ F_{4}\sin\left(\theta_{1}+\theta_{2}\right)=F_{3}\sin\theta_{1}+G\\ G=\rho \pi r^{2}gds_{2}\\ \frac{S_{2}}{2}F_{3}\sin\theta_{2}=J_{s}\ddot{\theta }\\ J_{s}=\frac{1}{12}\rho \pi r^{2}s_{2}^{3} \end{array}\right.,$$

Since ***F*_3_** = ***F*****_2_**, it follows that:

(39) $$F_{4}=\frac{\cos\theta_{1}}{\cos\left(\theta_{1}+\theta_{2}\right)}\left(F_{1}+\rho \pi r^{2}g\left(\mu \cos\theta_{1}+\sin\theta_{1}\right)S_{1}\right),$$

**Figure 14:**
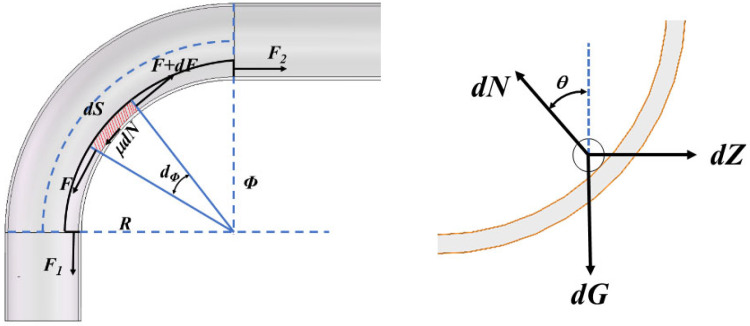
Force Analysis Diagram. Parameter Description: ***F*_1_** ***F*****_2_**—Tension forces at both ends of the cable, ***F***—Tension at lower end of segment, ***F*** + ***dF***—Tension at upper end of segment, ***dG***—Length of infinitesimal cable segment, ***dS***—Length of infinitesimal cable segment, ***μ***—Friction coefficient between cable and inner pipe wall, ***r***—Cable radius, ***dN***—Normal force acting on the infinitesimal cable segment, ***dZ***—Forces acting on the infinitesimal segment in the horizontal direction, ***Φ***—Bend-pipe radian, ***dΦ***—Infinitesimal-segment radian.

The stress condition of the cable at the curve is shown in Figure 14. The cable is closely attached to the inner wall of the curved pipe, with tensile forces ***F*_1_** and ***F*****_2_** acting on its two ends. An infinitesimal segment of the cable is selected for force analysis.

The length of the infinitesimal segment is ds. The infinitesimal segment is in contact with the inner wall of the pipe, subjected to a supporting force ***dN*** and a gravitational force ***dG***. The tensile forces acting on its two ends are F and ***F*** + ***dF***. ***R*** is the radius of curvature of the curved pipe, and ***Φ*** is the arc angle of the curved pipe.

From the force equilibrium conditions, it can be obtained that:

(40) $$\left\{\begin{array}{l} dZ=\sin\theta dN\\ \cos\theta dN=dG\\ dG=\rho \pi r^{2}gds\\ \sin\theta dN-\left(F+dF\right)\sin\frac{d\phi }{2}-F\sin\frac{d\phi }{2}=0\\ \left(F+dF\right)\cos\frac{d\phi }{2}-F\cos\frac{d\phi }{2}-\mu dN=0 \end{array}\right.,$$

Simplifying the equation yields:

(41) $$\left\{\begin{array}{l} \cos\theta dN=\rho \pi r^{2}gds\\ dF=\mu dN\;ds\\ \sin\theta dN=Fd\phi \end{array}\right.,$$

From Equations $\sin^{2}\alpha +\cos^{2}\alpha =1$ and ds=Rdϕ, it can be derived that:

(42) $$\left\{\begin{array}{l} dN=d\phi \sqrt{\rho^{2}\pi^{2}r^{4}g^{2}R^{2}+F^{2}}\\ dF=\mu d\phi \sqrt{\rho^{2}\pi^{2}r^{4}g^{2}R^{2}+F^{2}} \end{array}\right.,$$

Integrating the equation gives:

(43) $$\frac{F_{2}+\sqrt{F_{2}^{2}+\rho^{2}\pi^{2}r^{4}g^{2}R^{2}}}{F_{1}+\sqrt{F_{1}^{2}+\rho^{2}\pi^{2}r^{4}g^{2}R^{2}}}=e^{\mu \cdot \Delta \phi },$$

From the above-derived formulas, the following conclusion can be drawn: the cable towing resistance experienced by the pipeline robot when traveling in a curved pipe segment is only related to the arc angle ***Φ*** of the curved pipe and has no dependence on the radius of curvature ***R*** of the curved pipe itself. Further analysis of the correlation between towing resistance and arc angle ***Φ*** reveals that the cable resistance in curved pipe segments poses a significant obstacle to the robot’s movement, and this conclusion provides a key reference for the subsequent design optimization of the robot’s traction system.

### 3.5. Analysis of Inner Wall Grinding Operation

When the pipeline robot designed in this paper operates inside the pipeline, it adopts a motion mode coupling forward movement and rotary grinding, and its grinding heads can form a three-helix grinding trajectory on the inner wall of the pipeline. During this process, the grinding resistance not only hinders the robot’s forward movement but also exerts a rotational torque on the robot’s body, which in turn induces additional rotation of the body. This mechanical effect directly affects the motion stability and grinding trajectory accuracy of the robot. As shown in the diagram, a force analysis is performed on the pipeline robot during rotary grinding operations.

***F_n_*_1_**, ***F_n_*_2_**, and ***F_n_*_3_** represent the normal reaction forces exerted by the inner pipe wall on the ends of the three grinding devices, namely the tension forces of the grinding devices; ***F_f_*_1_**, ***F_f_*_2_**, and ***F_f_*_3_** denote the frictional forces between the ends of the three grinding devices and the inner pipe wall; ***θ*** is the angle between the velocity direction of the grinding device ends and the horizontal normal line of the pipeline axis; ***F*_1_**, ***F*_2_**, ***F*_3_**, and ***F*****_4_** are the supporting reaction forces exerted by the inner pipe wall on the four-wheel sets of the pipeline robot’s walking mechanism, namely the tension forces of the grinding devices.

**Figure 15:**
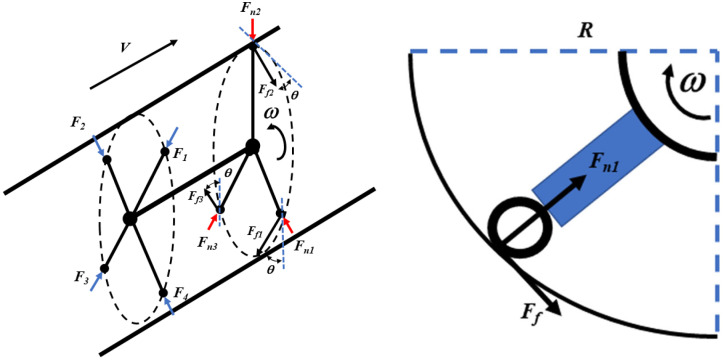
Dynamic Model of Grinding Operation. Parameter Description: ***F*_1_**–***F*_4_**—Reaction force on the walking-support module, ***F_f_*_1_**–***F_f_*_3_**—Friction force along the roller-steel-brush motion direction, ***θ***—Angle between the roller-steel-brush velocity direction and the normal to the pipe axis, ***R***—Pipe radius, ***V***—Direction of velocity, ***ω***—Rotational speed.

During the grinding operation of the robot, the robot is subjected to a reverse torque around the pipeline axis exerted by the inner pipe wall, and its force condition is illustrated in Figure 15.

The traction resistance generated by the grinding operation is expressed as:

(44) FZ=∑μsinθFni i=1,2,3,

The reverse torque ***M*** acting on a single grinding device is given by:

(45) $$M_{1}=RFf=\frac{\mu \cos\theta Fn1D}{2},$$

The total reverse torque acting on the robot body is calculated as:

(46) M=∑cosθRFfi=∑μcosθFniD2 i=1,2,3,

To ensure that the robot body does not deflect:

(47) $$\sum \frac{\mu DFi}{2}(i=1,2,3,4)\geq \sum \frac{\mu \cos\theta FniD}{2}(i=1,2,3),$$

That is:

(48) ∑Fi(i=1,2,3,4)≥∑cosθFni(i=1,2,3),

It can be clarified from the derived formulas that the traction resistance generated by the pipeline robot during grinding operations is mainly related to the included angle ***θ*** and the tension force of the grinding devices. The influence of these two factors on traction resistance exhibits a positive correlation: the larger the angle ***θ*** and the higher the tension force of the grinding devices, the greater the traction resistance acting on the robot. From the perspective of the robot body’s motion stability, to prevent the robot from deflecting during grinding operations, a key mechanical condition must be satisfied—specifically, the sum of the supporting reaction forces exerted by the inner pipe wall on the wheel sets of the traveling mechanism should be greater than the total tension force of each grinding device.

### 3.6. Chapter Summary

This section systematically examines the full-range motion characteristics of the proposed configuration within the pipe: a dynamic-coupling model for the collaborative force bearing of the four supporting mechanisms is established, the walking–support coupling mechanism is clarified, traction performance and tow-cable resistance constraints are quantified, and the dynamic disturbance law of the grinding load on the robot’s main-body attitude is emphasized. The resulting theoretical and experimental dataset forms a reusable design-evaluation framework that provides quantitative guidelines and a standardized analysis paradigm for the development of large-scale PCCP inner-wall grinding robots and similar equipment.

## 4. Performance Analysis of Pipeline Robots

### 4.1. Obstacle Crossing Performance Analysis

**Figure 16:**
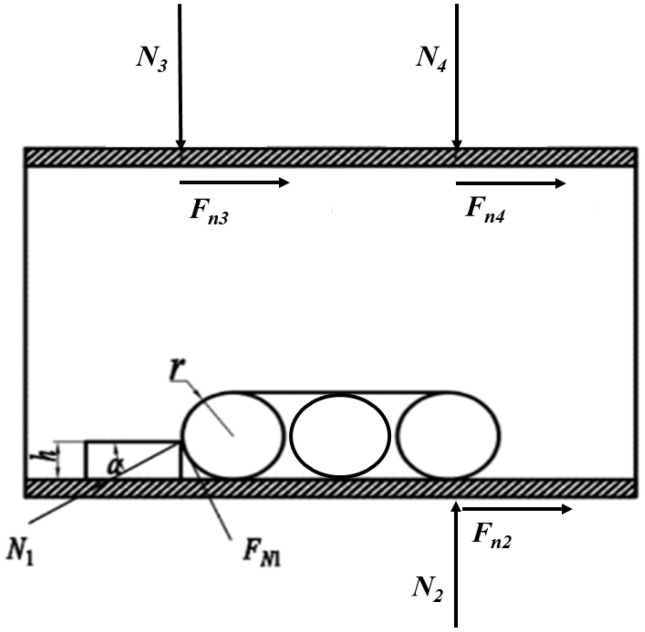
Obstacle Clearance Diagram. Parameter Description: ***N*_1_**–***N*_4_**—Normal (reaction) force, ***F_n_*_1_**–***F_n_*_4_**—Friction force, ***r***—Wheel radius, ***a***—Angle between the contact normal at the wheel–obstacle interface and the horizontal, ***h***—Obstacle height, ***ω***—Rotational speed, ***μ***—Friction coefficient between the wheel and the inner pipe wall, ***μ*_1_**—Friction coefficient between the wheel and the obstacle.

When a robot traverses obstacles (deposits, deformed protrusions) in pipelines, the robot’s obstacle-crossing height is a critical indicator of its obstacle-crossing capability [22,23]. The obstacle-crossing motion of a pipeline robot is simplified into the wheels crossing a rectangular block for analysis, as shown in Figure 16.

According to the static equilibrium equations and the geometric relationships in Figure 16, we have:

(49) $$\left\{\begin{array}{l} N_{1}[\cos\alpha -u_{1}\sin\alpha ]=u(N_{2}+N_{3}+N_{4})\\ N_{1}\sin\alpha +N_{1}u_{1}\cos\alpha +N_{2}=N_{3}+N_{4}\\ \sin\alpha =\frac{r-h}{r}\\ \cos\alpha =\frac{\sqrt{r^{2}-{\left(r-h\right)}^{2}}}{r} \end{array}\right.,$$

Combine and simplify using the above formulas

(50) $$h=r\left(1-\frac{1-uu_{1}}{\sqrt{\left(1+u^{2})(1+u_{1}^{2}\right)}}\right),$$

From Equation (50), it can be seen that the obstacle-surmounting height of the pipeline robot is related to the radius r of the supporting wheels, the friction coefficient μ between the supporting wheels and the inner wall of the pipeline, and the friction coefficient $\mu_{1}$ between the supporting wheels and obstacles. For this robot, the supporting wheels are designed with a radius of 62.5 mm, the friction coefficient between the supporting wheels and the inner pipeline wall is 0.5, and the friction coefficient between the supporting wheels and obstacles is 0.3. Through calculation, the maximum obstacle-surmounting height is found to be 17 mm.

### 4.2. Analysis of Cornering Performance

#### 4.2.1. Geometric Constraint Analysis

**Figure 17:**
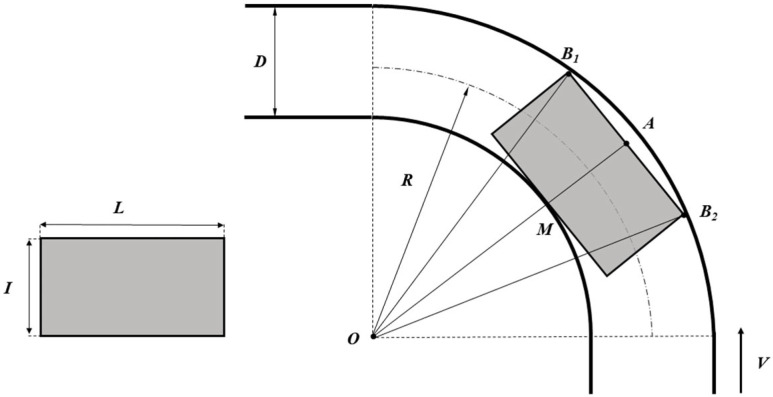
Diagram indicating a robot turning. Parameter description: ***L***—Axial length of the pipeline robot, ***I***—Width of the pipeline robot, ***D***—Pipe diameter, ***R***—Bend curvature radius.

Pipe robots are constrained by geometry when navigating bends; being too “short and thick” or “long and thin” can cause the robot’s external profile to interfere with the pipe wall, preventing passage. To address this issue, the pipe robot is simplified into a rigid unit for analysis, as shown in Figure 17 [24,25,26].

In the diagram, **R** denotes the curvature radius of the elbow, **D** is the pipe diameter, **L** represents the length of the robot, and I is the width of the pipeline robot. When the robot enters a curved section: If $OB_{2}>OB_{1}$, the contact arc trajectory constructed based on $OB_{2}$ will partially exceed the boundary of the pipe’s inner wall, leading to interference between the robot and the pipe wall during the turning process. If $OB_{2}\leq OB_{1}$, the contact arc trajectory constructed based on $OB_{2}$ will be entirely located inside the pipe’s inner wall during turning, meaning no interference occurs. Taking $OB_{2}=OB_{1}$ ($AB_{2}=AB_{2}$), according to the geometric relationships shown in Figure 17:

(51) $$\left\{\begin{array}{l} I⩽AM\\ AM=OA-OM\\ OA=\sqrt{OB_{2}^{2}-AB_{2}^{2}}=\sqrt{OB_{1}^{2}-AB_{1}^{2}}\\ OB_{2}=OB_{1}=R+D/2\\ OM=R-D/2 \end{array}\right.,$$

The solution is obtained

(52) $$\left\{\begin{array}{l} I⩽\sqrt{{\left(R+D/2\right)}^{2}-AB_{2}^{2}}-(R-D/2)\\ L⩽2\sqrt{{\left(2R+I\right)}^{2}(D-I)} \end{array}\right.,$$

**Figure 18:**
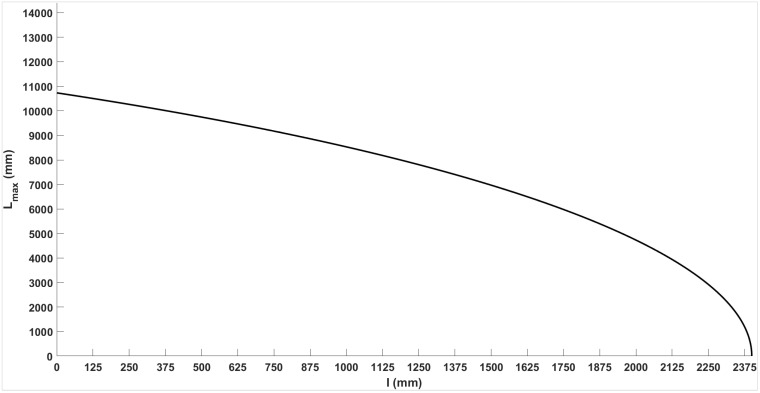
Robot limit length and width.

The axial and radial dimensions of the robot should satisfy Equation (52). Taking a pipe with a diameter D = 2400 mm and an elbow curvature radius of 2.5D as an example:

From Figure 18, it can be seen that the maximum allowable length of the pipeline robot decreases as the pipe diameter increases, and there is a non-linear inverse relationship between the maximum length and the pipe diameter. When the four supporting wheel sets of the robot expand to make contact with the inner pipe wall (with a width of I = 1679 mm), the maximum allowable length of the robot $L_{max}=6206\mathrm{mm}$ is obtained. Therefore, as long as the length of the pipeline robot does not exceed 6206 mm, it can pass through the pipe with a diameter D = 2400 mm and an elbow curvature radius of 2.5D.

#### 4.2.2. Transitional Phase Analysis

When the pipeline robot travels through an elbow, its motion can be divided into two phases: the transition phase and the rotation phase. In the transition phase, the front wheels of the robot enter the elbow, while the rear wheels remain in the straight pipe section [27,28,29,30,31].

**Figure 19:**
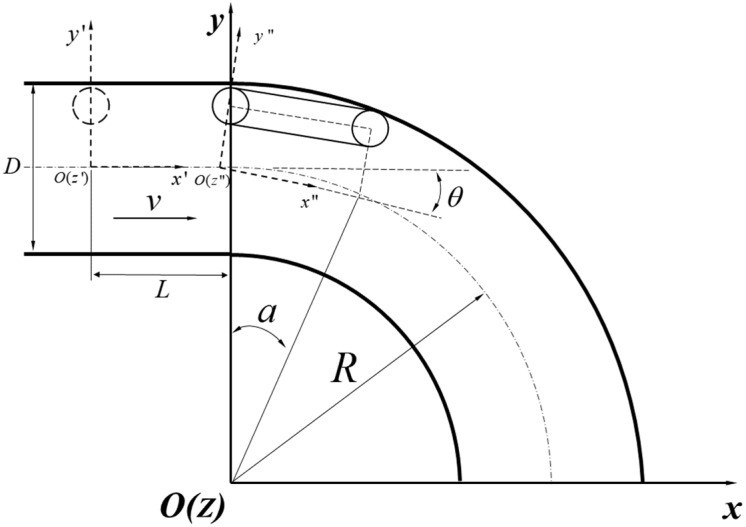
Transitional phase movement diagram. Parameter description: ***D***—Pipe diameter, ***R***—Bend curvature radius, ***a***—Rotation angle of the pipeline robot, ***θ***—Entry angle of the pipeline robot into the bend, ***L***—Wheelbase (distance between front and rear wheels), ***γ***—Attitude angle of the pipeline robot.

As shown in Figure 19, In the rotation phase, the robot is fully located within the elbow and moves by rotating around the curvature center of the elbow in space. A main coordinate system O−xyz and a slave coordinate system $O-x^{\prime }y^{\prime }z^{\prime }$ are established to analyze the motion state of the robot during the transition phase based on the planar motion of a rigid body.

The coordinates of the contact points between the front and rear wheels of the four-wheel set of the pipeline robot and the inner wall of the pipeline are as follows:

(53) FW′=L0.5Dcos45°+γ0.5Dsin45°+γL0.5Dcos135°+γ0.5Dsin135°+γ  L0.5Dcos225°+γ0.5Dsin225°+γL0.5Dcos315°+γ0.5Dsin315°+γ    ,

(54) RW′=00.5Dcos45°+γ0.5Dsin45°+γ00.5Dcos135°+γ0.5Dsin135°+γ  00.5Dcos225°+γ0.5Dsin225°+γ00.5Dcos315°+γ0.5Dsin315°+γ    ,

Equations (53) and (54) correspond to the trajectory matrices of the front wheels and rear wheels, respectively. Here, γ is the attitude angle, D is the pipe diameter, L is the distance between the front and rear wheels, α is the entry angle when entering the pipe, and θ is the rotation angle after entering the pipe. Then:

(55) Lsinθ=R(1−cosα),

The translational coordinates of the pipeline robot are as follows:

(56) $$P^{\prime }=\left[\begin{array}{c} R\sin\alpha -\sqrt{L^{2}-R^{2}{\left(1-\cos\alpha \right)}^{2}}\\ R\\ 0 \end{array}\right],$$

(57) $$R\sin\alpha -\sqrt{L^{2}-R^{2}{\left(1-\cos\alpha \right)}^{2}}=0,$$

It can reach a maximum equivalent to a bend angle.

(58) $$\alpha_{\max}=\arccos\left(1-\frac{L^{2}}{2R^{2}}\right),$$

(59) $$\theta =arc\tan\frac{R}{L-R\alpha }-arc\cos\frac{L^{2}+{\left(L-R\alpha \right)}^{2}}{2L\sqrt{R^{2}+{\left(L-R\alpha \right)}^{2}}},$$

Maximum Rotation Angle:

(60) $$\theta_{\max}=\arcsin\left(\frac{L}{2R}\right),$$

From this, it can be concluded that during the transition phase of the pipeline robot’s elbow traversal, the entry angle range is $0\leq \alpha \leq \alpha_{1}$, where $\alpha_{1}$ is related to L and R. Meanwhile, θ is determined by α, with its range being $0\leq \theta \leq \theta_{1}$. The contact points between the front/rear wheels of the wheel set and the inner pipe wall can be expressed in the main coordinate system as:

(61) $$Fw=Rot(Z^{\prime },-\theta )Fw^{\prime }+Trans,$$

(62) $$Rw=Rot(Z^{\prime },-\theta )Rw^{\prime }+Trans,$$

Here, Trans denotes the translation matrix, and Rot(Z′,−θ)Rot(Z,−θ) represents the rotation matrices.

(63) $$Trans=\left[\begin{array}{cccc} P^{\prime } & P^{\prime } & P^{\prime } & P^{\prime } \end{array}\right],$$

(64) $$Ror\left(Z^{\prime }-\theta \right)=\left[\begin{array}{ccc} \cos\left(\theta \right) & \sin\left(\theta \right) & 0\\ -\sin\left(\theta \right) & \cos\left(\theta \right) & 0\\ 0 & 0 & 1 \end{array}\right]$$

(65) $$\cos(\theta )=\frac{\sqrt{L^{2}-R^{2}{\left[1-\cos(\alpha )\right]}^{2}}}{L},$$

(66) $$\sin\left(\begin{array}{c} \theta \end{array}\right)=\frac{R}{L}\left[\begin{array}{c} 1-\cos(\alpha ) \end{array}\right]$$

(67) $$k_{1}=\cos(\theta ),$$

(68) $$k_{2}=\sin\left(\begin{array}{c} \theta \end{array}\right),$$

(69) $$k_{3}=R\sin(\alpha )-L\cos(\theta ),$$

The trajectory equations of the front and rear wheels can be expressed as:

(70) FW=Rsinα+0.5k2Dcos45°+γRcosα+0.5k1Dcos45°+γ0.5Dsin45°+γRsinα+0.5k2Dcos135°+γRcosα+0.5k1Dcos135°+γ0.5Dsin135°+γ  Rsinα+0.5k2Dcos225°+γRcosα+0.5k1Dcos225°+γ0.5Dsin225°+γRsinα+0.5k2Dcos315°+γRcosα+0.5k1Dcos315°+γ0.5Dsin315°+γ    ,

(71) RW=0.5Dcos(45°+γ)k2+k30.5Dcos(45°+γ)k1+R0.5Dsin(45°+γ)0.5Dcos135°+γk2+k30.5Dcos135°+γk1+R0.5Dsin135°+γ  0.5Dcos225°+γk2+k30.5Dcos225°+γk1+R0.5Dsin225°+γ0.5Dcos315°+γk2+k30.5Dcos315°+γk1+R0.5Dsin315°+γ     ,

To obtain the velocity expressions of the front and rear wheels during the transition phase, we take the time derivative of the trajectory equations (Equations (70) and (71)) for the front and rear wheels of the wheel set, respectively:

(72) $$\frac{dFw}{dt}=\left[\begin{array}{cccc} \nu_{fx1} & \nu_{fx2} & \nu_{fx3} & \nu_{fx4}\\ \nu_{fy1} & \nu_{fy2} & \nu_{fy3} & \nu_{fy4}\\ 0 & 0 & 0 & 0 \end{array}\right],$$

(73) $$\nu_{fxi}=R\dot{\alpha }\left\{\cos\left(\alpha \right)+\frac{0.5D}{L}\cos[\gamma +\left(i\right)45^{{}^{\circ}}]\sin\left(\alpha \right)\right\}i=1,2,3,4$$

(74) $$\nu_{fyi}=-R\sin\left(\alpha \right)\dot{\alpha }\left\{1+\frac{0.5D\cos\left[\gamma +\left(i\right)45^{{}^{\circ}}\right]R\left[1-\cos\left(\alpha \right)\right]}{L\sqrt{L^{2}-R^{2}{\left[1-\cos\left(\alpha \right)\right]}^{2}}}\right\}i=1,2,3,4,$$

(75) $$\frac{dRw}{dt}=\left[\begin{array}{cccc} \nu_{rx1} & \nu_{rx2} & \nu_{rx3} & \nu_{rx4}\\ \nu_{ry1} & \nu_{ry2} & \nu_{ry3} & \nu_{ry4}\\ 0 & 0 & 0 & 0 \end{array}\right]$$

(76) $$\nu_{rxi}=R\dot{\alpha }\left\{\cos\left(\alpha \right)+\frac{R\sin\left(\alpha \right)\left[1-\cos\left(\alpha \right)\right]}{L\sqrt{L^{2}-R^{2}{\left[1-\cos\left(\alpha \right)\right]}^{2}}}+\frac{0.5D}{L}\cos\left[\gamma +\left(i\right)45^{{}^{\circ}}\right]\sin\left(\alpha \right)\right\}i=1,2,3,4,$$

(77) νryi=−0.5Dcosγ+i45°R2sinα1−cosαLL2−R21−cosα2α  i=1,2,3,4,

The speeds of the front and rear wheels of the pipeline robot can be expressed as:

(78) $$V_{fi}=\sqrt{v_{fxi}^{2}+v_{fyi}^{2}},$$

(79) $$V_{ri}=\sqrt{v_{rxi}^{2}+v_{ryi}^{2}},$$

Based on Equations (40) and (41), the theoretical speed values for each wheel set during the robot’s transition phase can be computed. The rotational speed ratios of the individual wheels are:

(80) $$V_{r1}:V_{r2}:V_{r3}:V_{r4}:V_{r1}:V_{r2}:V_{r3}:V_{r4}=n_{r1}:n_{r2}:n_{r3}:n_{r4}:n_{r1}:n_{r2}:n_{r3}:n_{r4},$$

#### 4.2.3. Rotational Phase Analysis

**Figure 20:**
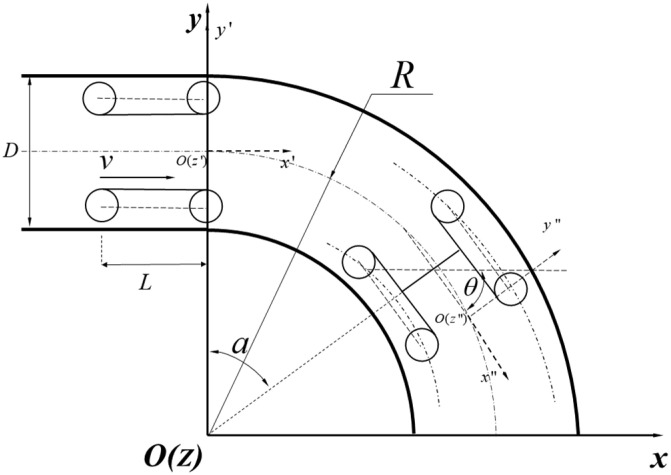
Motion diagram of the rotating phase. Parameter Description: ***D***—Pipe diameter, R—Bend curvature radius, ***a***—Rotation angle of the pipeline robot, ***θ***—Entry angle of the pipeline robot into the bend, ***L***—Wheelbase (distance between front and rear wheels), ***γ***—Attitude angle of the pipeline robot.

The pipeline robot fully enters the bend of the pipe and rotates around the center of curvature of the bend. According to Figure 20, the contact coordinates of the front and rear wheels of the pipeline robot with the inner wall of the pipe can be expressed as:

(81) Fw=xf1xf2yf1yf20.5Dsin45°+γ0.5Dsin135°+γ  xf3xf4yf3yf40.5Dsin225°+γ0.5Dsin315°+γ    ,

Front Wheel Coordinates $X_{fi}$, $Y_{fi}$, $Z_{fi}$

(82) $$\left\{\begin{array}{c} X_{fi}=R\sin\alpha +0.5\sin\alpha \sin(\gamma +45^{{}^{\circ}}i)D\\ Y_{fi}=R\cos\alpha +0.5\cos\alpha \sin(\gamma +45^{{}^{\circ}}i)D\\ Z_{fi}=0.5D\sin(\gamma +45^{{}^{\circ}}i)\begin{array}{c} \;\;\;\;i=1,2,3,\;4 \end{array} \end{array}\right.,$$

(83) $$Rw=\left[\begin{array}{cc} x_{f1}-L\cos\alpha & x_{f2}-L\cos\alpha \\ y_{f1}+L\sin\alpha & y_{f2}+L\sin\alpha \\ 0.5D\sin\left(45^{{}^{\circ}}+\gamma \right) & 0.5D\sin\left(135^{{}^{\circ}}+\gamma \right) \end{array}\right.\begin{array}{cc} x_{f3}-L\cos\alpha & x_{f4}-L\cos\alpha \\ y_{f3}+L\sin\alpha & y_{f4}+L\sin\alpha \\ 0.5D\sin\left(225^{{}^{\circ}}+\gamma \right) & 0.5D\sin\left(315^{{}^{\circ}}+\gamma \right) \end{array}\left.\right],$$

The coordinates of the rear wheel can be expressed as:

(84) $$\left\{\begin{array}{c} X_{fi}=R\sin\alpha +0.5\sin\alpha \sin(\gamma +45^{{}^{\circ}}i)D\\ Y_{fi}=R\cos\alpha +0.5\cos\alpha \sin(\gamma +45^{{}^{\circ}}i)D\\ Z_{fi}=0.5D\sin(\gamma +45^{{}^{\circ}}i)\;\;\;\;i=1,2,3,4 \end{array}\right.,$$

The front- and rear-wheel speeds of the pipeline robot can be expressed as:

(85) $$V_{fi}=\sqrt{v_{fxi}^{2}+v_{fyi}^{2}},$$

(86) $$V_{ri}=\sqrt{v_{rxi}^{2}+v_{ryi}^{2}},$$

Equations (85) and (86) describe the trajectory of the robot during the rotary phase of elbow negotiation. At this stage, the ratio of the wheel speeds equals the ratio of their respective path–curvature radii—i.e., the bend–curvature radii—so the wheel–speed ratio of the robot is:

(87) $$n_{f1}:n_{f2}:n_{f3}:n_{f4}:n_{r1}:n_{r2}:n_{r3}:n_{r4}=R_{f1}:R_{f2}:R_{f3}:R_{f4}:R_{r1}:R_{r2}:R_{r3}:R_{r4},$$

#### 4.2.4. Polishing Path Planning

**Figure 21:**
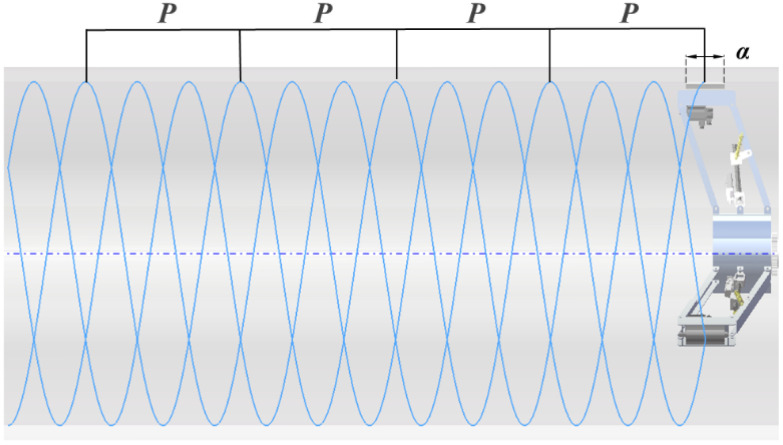
Polishing Track. Parameter description: ***a***—Steel-brush length, ***P***—Helical pitch (general), ***P_inter_***—Outer-helical pitch, ***P_outer_***—Inner-helical pitch, ***D***—Pipe diameter, ***R***—Bend curvature radius.

When the grinding head rotates, the steel brushes create a helical pattern on the inner wall of the pipe, as shown in Figure 21. To ensure that the three cylindrical steel brushes fully cover the pipe’s inner wall as the robot moves forward, it is necessary to adjust the relationship between the rotational speed and the traveling speed.

The three steel brushes are spaced 120° apart. When the robot completes one full rotation, the helical trajectory of each steel brush is separated by *P*/3 in the axial direction. To ensure full axial coverage, the trajectories of adjacent steel brushes must satisfy:

(88) $$\frac{P}{3}\leq a\;\Rightarrow \;P\leq 3a\;\Rightarrow \;\omega \geq \frac{2\pi v}{3a},$$

Here, ***P*** is the pitch of a single spiral, ***a*** denotes the length of the steel brush, and ***V*** is the forward speed of the pipeline robot.

**Figure 22:**
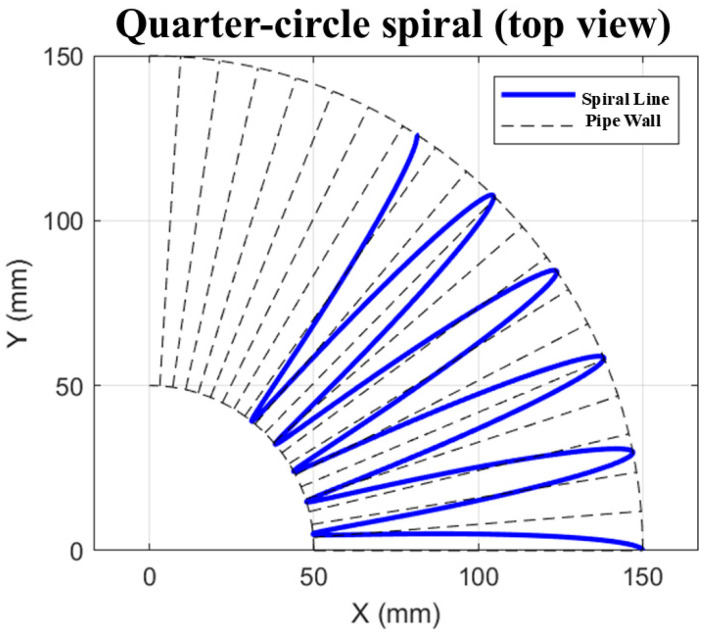
Bending pipe polishing trajectory.

When operating in an elbow (where the pipe axis follows an arc), if the robot maintains a constant speed along the arc trajectory, the helical grinding trajectories will exhibit a distribution pattern of being sparse on the outer side and dense on the inner side of the elbow (as shown in Figure 22). This is because the path lengths of the inner and outer sides of the pipe differ. At this point, the full-coverage condition is: the outermost spiral pitch must not exceed the length of the steel brush. Since the steel brush is much smaller than the pipe dimensions, the contact error between them can be neglected.

The pitch on the inner and outer sides of the curve can be expressed as:

(89) $$P_{\mathrm{inner}}=P\frac{R-D/2}{R},$$

(90) $$P_{\mathrm{outer}}=P\frac{R+D/2}{R},$$

***Pinner*** refers to the spiral pitch on the inner side of the pipe, ***Pouter*** is the spiral pitch on the outer side of the pipe, ***P*** denotes the spiral pitch along the pipe axis direction, ***D*** is the pipe diameter, and *R* is the curvature radius of the pipe.

(91) $$P=\frac{2\pi v}{\omega },$$

(92) $$P_{\mathrm{outer}}\leq a=\frac{P(R+D/2)}{3R}\leq a,$$

Substituting Equation (91) into Equation (92) yields:

(93) $$\omega \geq \frac{2\pi v}{3a}\cdot \frac{R+D/2}{R},$$

In practical applications, it is recommended to select a value of **1.2 *ω*** (with a safety margin reserved) to prevent missed grinding caused by mechanical vibration or slippage.

### 4.3. Chapter Summary

This chapter presents a systematic analysis of the mobility of our newly designed in-pipe robot. Geometric constraints for obstacle crossing, the two-phase kinematics of elbow negotiation (transition and rotation), and full-coverage grinding paths are all examined, yielding an integrated locomotion-and-operation model. Together, these findings provide a complete picture of the robot’s motion in representative pipe environments and furnish a ready-to-use theoretical basis for future work on trajectory planning, motion control, and intelligent grinding adaptation.

## 5. Research on Simulation and Experimental Study of Pipeline Robots

After theoretically analyzing the various motions of the pipeline robot during its movement within the pipeline, in order to verify the robot’s passage capability and the rationality of its design, a three-dimensional model was created in SOLIDWORKS2022. After simplification, it was imported into Adams for simulation and verification analysis. Material parameters were set in Adams as shown in Table 1.

### 5.1. Analysis of Cornering Performance

**Figure 23:**
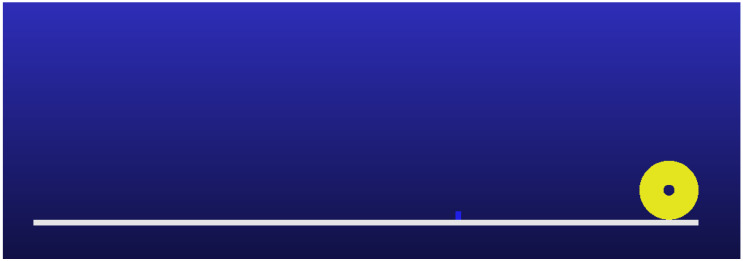
Wheel obstacle crossing simulation.

To verify the validity of the theoretical analysis on obstacle negotiation of the pipeline robot presented in Section 4.1, a simplified obstacle negotiation simulation model for the wheel set is established in this subsection (as shown in the Figure 23) for targeted analysis. A fixed joint is created between the ground plane and the global frame, and a constant rotational speed—updated in real time with the wheel’s physical motion—is applied to the driving wheel. Solid-to-solid contacts are defined between the wheel and both the fixed plane and the obstacle: the friction coefficient is set to 0.5 for the wheel–plane interface and 0.3 for the wheel–obstacle interface; all remaining parameters retain their default values. The solver is configured to use the stiff-capable GSTIFF integrator with a third-order implicit I3 formulation. Employing the original correction strategy and an adaptive step-size controlled by a local truncation error tolerance of 1.0 × 10^−3^, the simulation proceeds without an imposed maximum step size, ensuring both numerical stability and computational efficiency. To investigate the influence of wheel diameter on obstacle negotiation performance, three obstacle negotiation models of driving wheels with different specifications (radii of 40 mm, 60 mm, and 80 mm, respectively) are established for comparative analysis.

The maximum obstacle-surmounting heights of the driving wheels with three different sizes can be calculated according to the formulas presented in Section 4.1, and the results are listed in Table 2.

**Figure 24:**
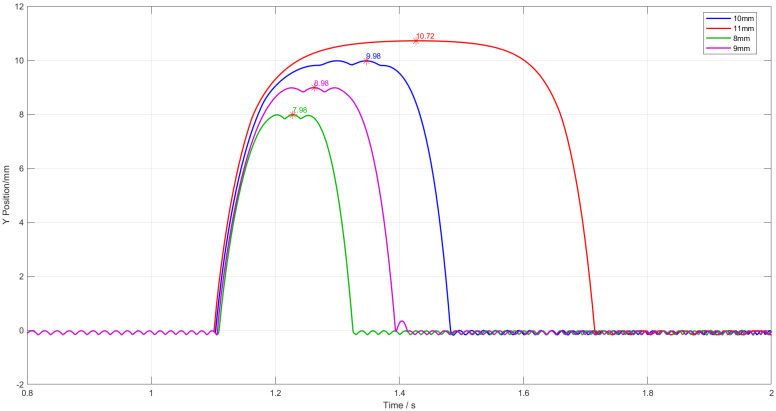
Change in the center of mass position of a 40 mm radius wheel.

A simulation analysis was conducted on the obstacle negotiation performance of the driving wheel with a radius of 40 mm over rectangular protrusion obstacles of different heights, where the obstacle heights were set to 8 mm, 9 mm, 10 mm, and 11 mm, respectively. The simulation results show that this type of driving wheel can successfully surmount obstacles with heights of 8 mm, 9 mm, and 10 mm but fails to cross the 11 mm-high obstacle. To further analyze the motion characteristics of the driving wheel during obstacle negotiation, the position coordinates of the driving wheel’s centroid in the Y-direction derived from the simulation were analyzed. The starting point of the Y-direction position curve of the driving wheel was corrected to the origin of the coordinate system so as to more intuitively observe the variation law of the driving wheel’s position in the Y-direction during obstacle negotiation. It can be seen from the processed simulation result diagram (Figure 24) that when the driving wheel surmounts obstacles of 8 mm, 9 mm, 10 mm, and 11 mm heights, the time consumed for obstacle negotiation shows an increasing trend with the increase of obstacle height, indicating that the higher the obstacle is, the greater the difficulty of obstacle negotiation for the driving wheel. Specifically, when crossing the 11 mm-high obstacle, the maximum lifting height of the driving wheel’s centroid is only 10.72 mm, which not only fails to reach the 11 mm obstacle height but also does not meet the theoretical maximum height of 10.87 mm, resulting in the failure of obstacle negotiation. When successfully surmounting obstacles of 8 mm, 9 mm, and 10 mm heights, there is a deviation of 0.02 mm between the maximum centroid height of the driving wheel and the corresponding obstacle height.

**Figure 25:**
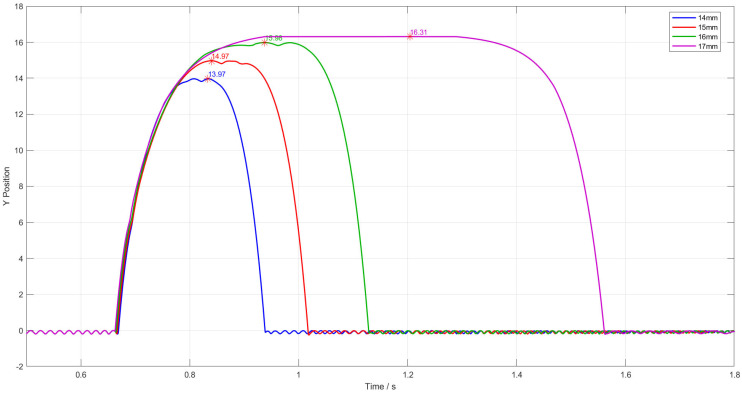
Change in the center of mass position of a 60 mm radius wheel.

As shown in Figure 25, According to the theoretical calculation results, the maximum obstacle-surmounting height of the driving wheel with a radius of 60 mm is approximately 16.31 mm. A simulation analysis on obstacle negotiation was carried out for this driving wheel against obstacles of four different heights (14 mm, 15 mm, 16 mm, and 17 mm), and the position variation curves of the driving wheel’s centroid in the Y-direction under various working conditions were obtained and sorted out (as shown in the diagram). The simulation results show that this driving wheel can successfully surmount obstacles with heights of 14 mm, 15 mm, and 16 mm but fails to cross the 17 mm-high obstacle: the maximum lifting height of its centroid is about 16.31 mm, after which it remains stagnant for a relatively long time and still cannot be lifted further to surmount the obstacle, eventually resulting in obstacle negotiation failure.

**Figure 26:**
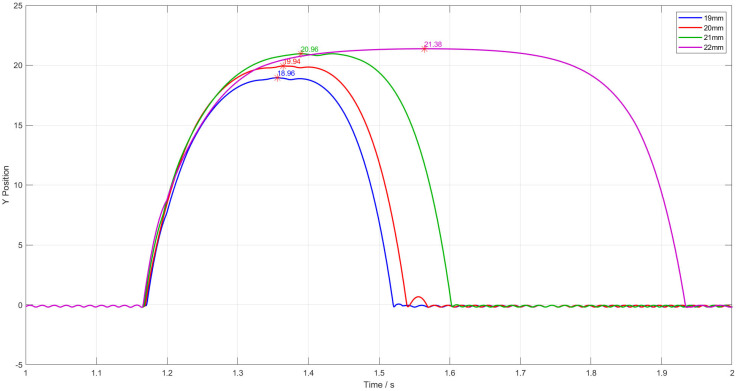
Change in the center of mass position of an 80 mm radius wheel.

As shown in Figure 26, According to the theoretical calculation results, the maximum obstacle-surmounting height of the driving wheel with a radius of 80 mm is approximately 21.74 mm. A simulation analysis on obstacle negotiation was conducted for this type of driving wheel against obstacles of four different heights, namely 19 mm, 20 mm, 21 mm, and 22 mm. The simulation results show that this driving wheel can successfully surmount obstacles with heights of 19 mm, 20 mm, and 21 mm; however, when attempting to cross the 22 mm-high obstacle, the maximum lifting height of its centroid only reaches 21.38 mm, which fails to meet the obstacle height. After a period of near-stagnant movement, the driving wheel ultimately fails to negotiate the obstacle.

The Summary of Simulation Experimental Results for the three-wheel specifications is summarized in Table 3. The obstacle negotiation simulation results of the three driving wheels with different specifications verify the correctness of the obstacle negotiation theoretical model established in Section 4.1. By synthesizing the obstacle negotiation performance data of the three driving wheel specifications, the following motion law can be summarized: when a driving wheel surmounts an obstacle, the time consumed for obstacle negotiation increases with the rise of obstacle height. This phenomenon indirectly confirms that the higher the obstacle is, the greater the difficulty of obstacle negotiation for the driving wheel. In particular, when the obstacle height is close to the theoretical maximum obstacle-surmounting height, the time consumed for obstacle negotiation will be significantly prolonged, and the hindering effect during the obstacle negotiation process will become more pronounced.

**Figure 27:**
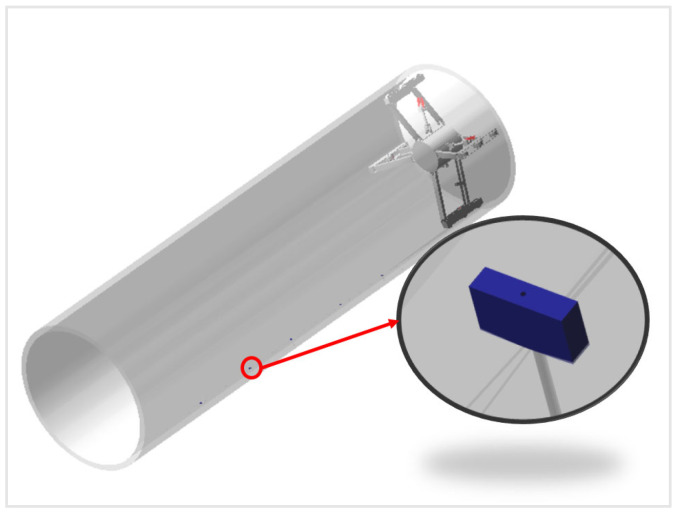
Obstacle simulation diagram.

Combined with the results of on-site investigations, the maximum height of protrusion obstacles detected on the inner wall of actual PCCP pipelines is approximately 14 mm. To ensure the robot has an adequate safety margin during operation, this study selects a value 1.2 times the actual maximum obstacle height as the analysis basis. In the design phase of the pipeline robot, the radius of the motor wheel hub to be adopted is set at 62.5 mm, and theoretical calculations show that its maximum obstacle-surmounting height is approximately 17 mm. To verify and analyze the actual obstacle-surmounting capability of the robot, an obstacle-surmounting simulation model of the pipeline robot was established using Adams2020. Rectangular protrusion obstacles with five different heights (10 mm, 12 mm, 15 mm, 17 mm, and 20 mm) were successively arranged on the inner wall of the pipeline, and the specific model is shown in Figure 27.

In the simulation model, the pipe is rigidly fixed to the ground via a fixed joint, while revolute joints constrain all relatively moving links; since diameter adaptation is not considered, the lead screw and nut are also locked by a fixed joint. A constant rotational speed, updated in real time with the wheel’s physical motion, is applied to the driving wheel. Solid-to-solid contacts are defined between the wheel and both the fixed plane and the obstacle: the friction coefficient is set to 0.5 for the wheel–plane interface and 0.3 for the wheel–obstacle interface; all remaining parameters retain their default values. The solver is configured to use the stiff-capable GSTIFF integrator with a third-order implicit I3 formulation. Employing the original correction strategy and an adaptive step-size controlled by a local truncation error tolerance of 1.0 × 10^−3^, the simulation proceeds without an imposed maximum step size, ensuring both numerical stability and computational efficiency.

After the simulation starts, the robot can pass through obstacles of 10 mm, 12 mm, 15 mm, and 17 mm smoothly but fails to traverse the 20 mm raised obstacle.

**Figure 28:**
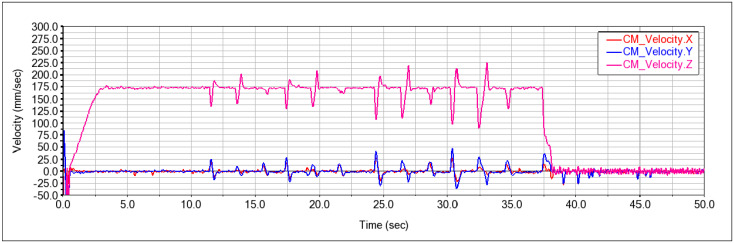
Centroid velocity.

**Figure 29:**
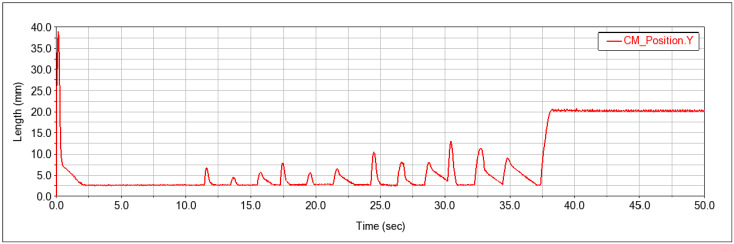
Centroid position.

Figure 28 shows the centroid velocity of the pipeline robot during the obstacle-surmounting simulation: After a period of acceleration, the robot’s velocity stabilizes at 170 mm/s. When the three wheels of the wheel set cross an obstacle, the velocity in the forward Z-direction drops sharply, while the velocity in the vertical Y-direction spikes. When attempting to cross the 20 mm obstacle, the Z-direction velocity oscillates around 0; The Y-direction velocity rises slightly, then oscillates several times before stabilizing at 0. The curve of the robot’s centroid position in the Y-direction during obstacle surmounting is shown in Figure 29: When the wheel set crosses an obstacle, the centroid position moves upward three times. However, when traversing the 20 mm raised obstacle, the centroid gets stuck 20 mm later, and its position no longer changes.

It can thus be seen that the pipeline robot fails to surmount the 20 mm raised obstacle, which verifies the correctness of the theoretical analysis in Section 4.1.

By establishing an integrated virtual prototype model of the pipeline robot, a simulation analysis was carried out on the entire process of its obstacle negotiation. The simulation results show that the actual maximum obstacle-surmounting height of the robot is basically consistent with the theoretical calculation value, which verifies the correctness and reliability of the theoretical analysis presented earlier. Meanwhile, the conclusions drawn from the simulation analysis in this study also provide practical theoretical guidance and data support for the subsequent selection of key components of the pipeline robot and the design optimization to enhance the robot’s adaptability to pipelines under different working conditions.

### 5.2. Simulation Analysis of Curve Traffic

**Figure 30:**
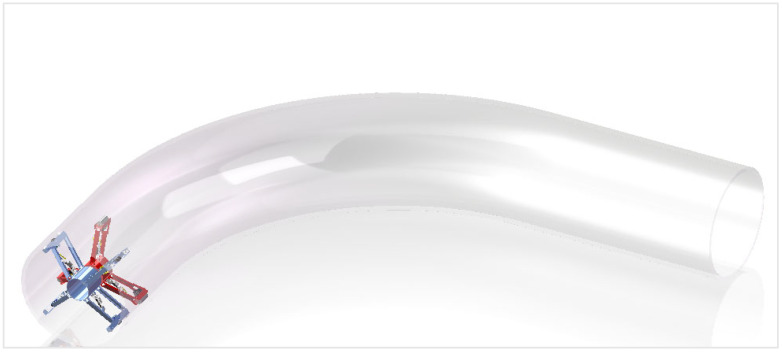
Pipeline robot curve navigation simulation.

This subsection presents a simulation analysis of the robot negotiating a bend for a pipe of a specific size: the inner diameter is chosen according to actual operating conditions, and the bend radius is set to 2.5 D. The robot’s main body measures 1 120 mm in length and 550 mm in diameter—dimensions that satisfy the geometric-constraint theory outlined in Section 4.2.1. The virtual prototype of the pipe robot used for the bend-passing simulation is shown in Figure 30.

**Figure 31:**
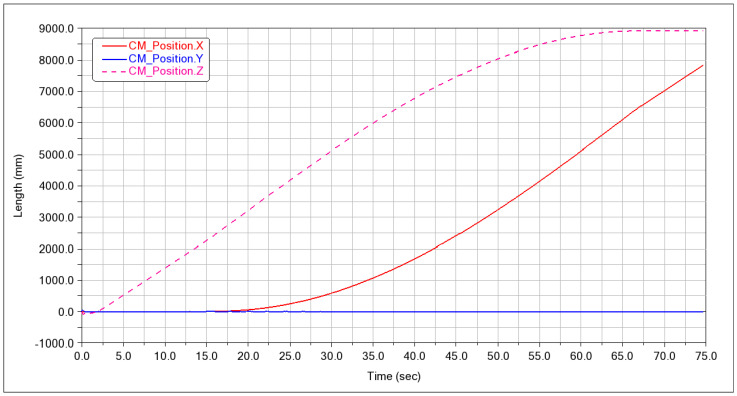
Centroid position.

**Figure 32:**
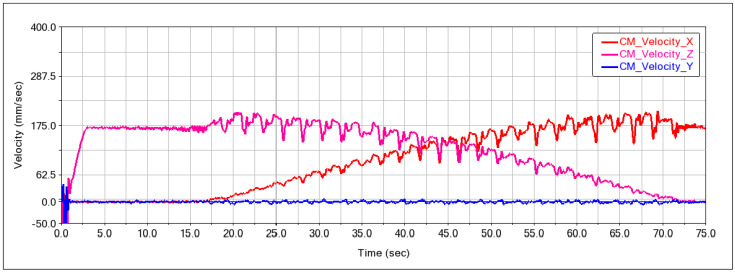
Centroid velocity.

In the ADAMS environment, the driving wheels were assigned the motion function STEP (time,0,0,3,120d), and the contact friction coefficient between the wheel sets and the pipe inner wall was set to 0.5. The study focused on how the robot’s centroid position and centroid velocity evolve during the bend negotiation. Figure 31 and Figure 32 show that, in the initial phase, the robot accelerates along the *Z*-axis in the straight section and then stabilizes at 175 mm s^−1^. Within the first 15 s, displacement of the centroid occurs only in the Z direction; no appreciable change is observed in X or Y. At t = 15 s, a displacement in X appears, indicating entry into the curved section. Once inside the bend, the centroid continues to move in both X and Z, whereas the Y coordinate (vertical) remains constant. Correspondingly, the centroid velocity exhibits an oscillatory rise in X and an oscillatory fall in Z. Around t = 66 s, the Z-position of the centroid levels off, while the X-position keeps increasing; the X-velocity gradually ceases its oscillatory rise and becomes steady, and the Z-velocity oscillates downward to nearly zero. At this point, the robot has successfully negotiated the bend without jamming, confirming that the proposed design offers excellent curved-pipe traversability.

As discussed in Section 4.2.3, the contact points of the individual driving wheels with the pipe wall differ during bend negotiation, so their trajectories are markedly dissimilar; consequently, each wheel set must be supplied with a distinct rotational speed to meet the demands of curved travel.

**Figure 33:**
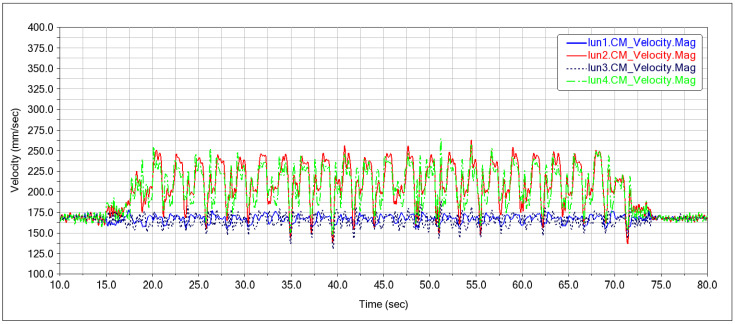
Wheel centroid speed.

To examine the robot’s behavior when a single, uniform-speed control policy is used, all wheels were given the same angular velocity in this simulation. Figure 33 compares the centroid–velocity histories of the inner wheel sets (1 and 3) and the outer wheel sets (2 and 4). The results show a pronounced speed discrepancy: the average velocity ratio of inner to outer sets is approximately 1:1.3, and the outer sets exhibit far larger fluctuations. This mismatch not only introduces additional internal losses but also excites severe body vibration, degrading overall running stability.

Building on the conclusions of Section 4.2.3, the wheel sets of the pipe robot follow different trajectories while negotiating a bend, so each set requires a distinct angular velocity.

**Figure 34:**
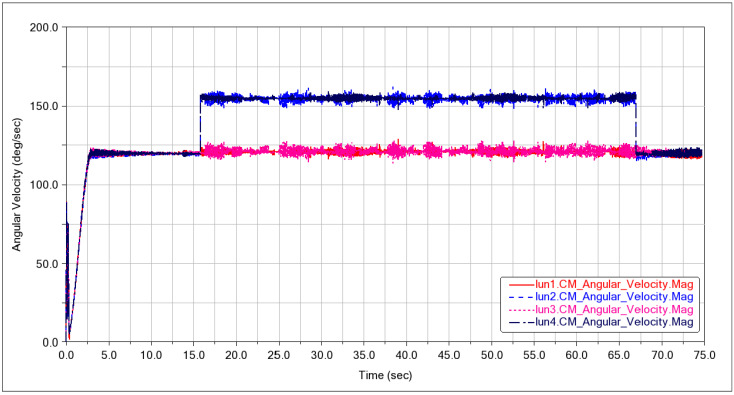
Wheel angular velocity.

Using the 1:1.3 average-speed ratio obtained from the earlier same-speed simulation, we assigned an angular-velocity ratio of 1:1.3 to the inner and outer wheel sets during bend traversal: 120° s^−1^ for the inner sets (1 and 3) and 156° s^−1^ for the outer sets (2 and 4), as shown in Figure 34. The robot’s bending performance under this differential-speed strategy was then compared with that under the uniform-speed strategy.

**Figure 35:**
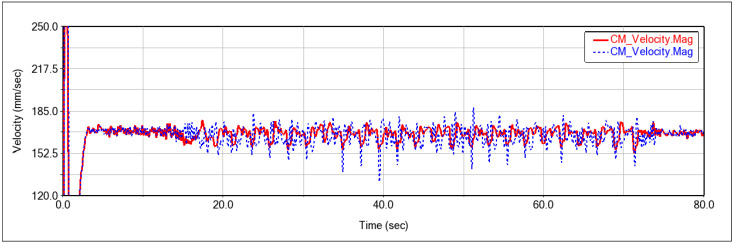
Center of mass velocity.

The centroid–velocity curves for the two cases (Figure 35) reveal that the differential-speed control (red solid line) produces markedly smaller velocity fluctuations and a smoother running speed than the uniform-speed control (blue dotted line).

**Figure 36:**
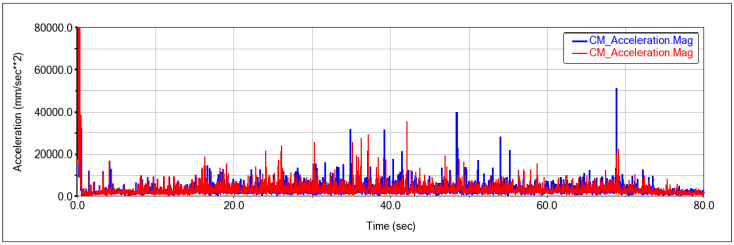
Acceleration of the body’s center of mass vibrations.

A further comparison of centroid accelerations (Figure 36) shows that the body vibration acceleration under differential-speed control (red solid line) is both lower in magnitude and smoother throughout the bend, whereas the uniform-speed case (blue curve) exhibits larger, more erratic oscillations.

### 5.3. Polishing Path Simulation Analysis

**Figure 37:**
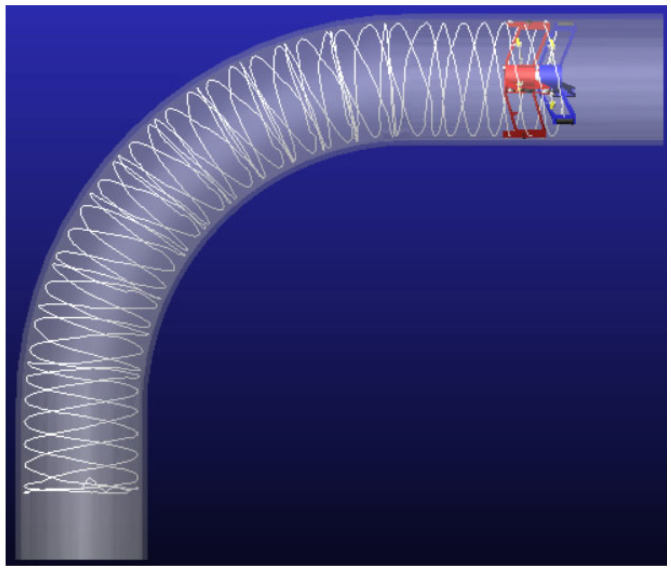
Olishing track.

Building on the simulation setup in Section 5.2, a grinding-head orbital speed of 0.47 rad/s—derived from the theoretical analysis in Section 4.2.4—was imposed. Figure 37 shows the resulting grinding traces: in the straight section, the three helical paths are regular and evenly overlapped, whereas in the bend, they become non-uniform; local speed fluctuations make the helices jitter, but the overall pattern still matches the prediction—dense on the inner side and sparse on the outer side.

**Figure 38:**
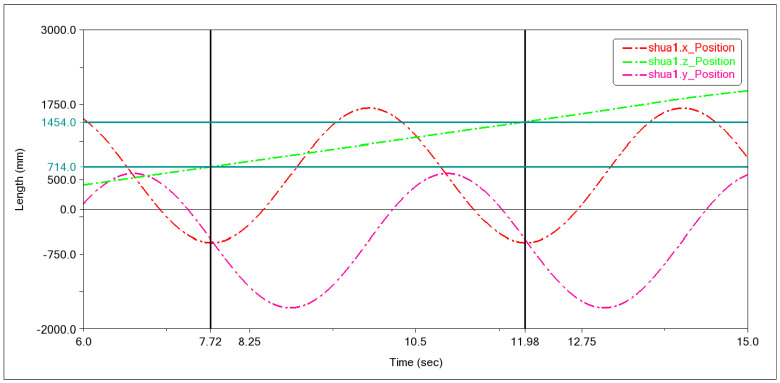
Olishing steel brush position curve diagram.

When the robot grinds in a straight pipe, the three helical traces are highly regular, so one complete revolution of the grinding head is selected for analysis. As shown in Figure 38, while the steel brush orbits once, the robot advances 740 mm along the *Z*-axis. According to Section 4.2.4, full coverage of the inner wall by the triple-helix pattern requires that one-third of a single helix lead be shorter than the brush length. The simulation gives 740 mm/3 = 246 mm, which is less than the 300 mm brush length; hence, the result satisfies the analysis in Section 4.2.4.

**Figure 39:**
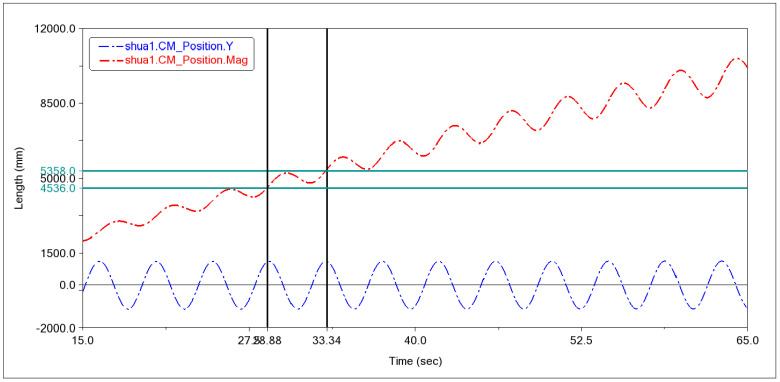
Olishing steel brush position curve diagram.

When the robot grinds inside an elbow, the three helical traces fluctuate markedly; the revolution with the largest brush travel is, therefore, selected for analysis. As shown in Figure 39, one complete orbit of brush “shua1” produces a total displacement of 822 mm. For full coverage in a bend, the triple-helix condition requires that one-third of the pitch of the outermost helix be shorter than the brush length. One-third of 822 mm is 274 mm, which is again less than the 300 mm brush length, so the requirement is satisfied.

The simulation results confirm that the robot’s grinding pattern aligns with the analysis in Section 4.2.2 and validates the full-coverage conditions for the inner pipe wall.

### 5.4. Chapter Summary

Based on the theoretical derivations presented in Section 5, this chapter establishes a virtual prototype of the in-pipe grinding robot in ADAMS and conducts motion simulations for three critical tasks: obstacle crossing, elbow negotiation, and grinding-path tracking. The simulation parameters are taken directly from the boundary conditions of the theoretical model, and the results match the analytical predictions, confirming the validity of the derived equations. The simulations also visualize the robot’s posture and force history within complex pipe segments, providing a comprehensive picture of the mechanism’s motion behavior and demonstrating the feasibility and rationality of the proposed design.

## 6. Experimental Verification and Conclusions

### 6.1. Experimental Verification

**Figure 40:**
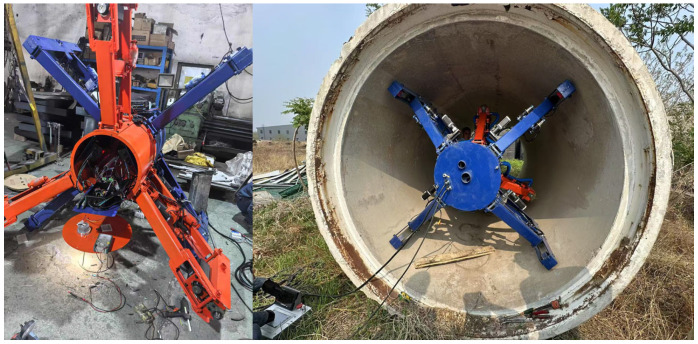
Ipeline robot prototype and experiment.

Based on the theoretical analysis and simulation verification of the pipeline inner-wall polishing robot, the preliminary preparation of the pipeline robot prototype has been completed, as shown in Figure 40. The body is made from 6 mm steel plates, which are cut, bent, and welded. The cylindrical main body has a diameter of 550 mm and is fully detachable, allowing it to pass through an 800 mm exhaust valve to enter the pipeline for reassembly. According to the functional requirements of the pipeline robot, the preliminary PLC-based control program has been developed. Through debugging, the pipeline robot is now capable of performing a series of basic actions, including moving forward and backward, supporting diameter adjustment, and forwarding and reversing polishing.

Inner wall grinding is a crucial preprocessing step in the repair and reinforcement projects of Prestressed Concrete Cylinder Pipes (PCCP), and its construction quality directly affects the subsequent repair and reinforcement effect. Currently, PCCP pipeline repair is mainly divided into two categories: open-cut repair and trenchless repair. Open-cut repair requires excavating the ground surface to expose the pipeline, followed by repair operations or direct pipe replacement; trenchless repair is mainly applied in scenarios where there are buildings or structures on the ground surface above the pipeline, or where the excavation is extremely difficult due to complex geological conditions [32,33].

The robot studied in this paper is specifically designed for inner wall grinding operations in the trenchless repair of large-diameter PCCP pipelines. Compared with traditional pipeline inspection robots and other similar equipment, this grinding robot features a large size and significantly increased self-weight, which makes the feasibility of simulating and constructing a pipeline experimental environment extremely low. In addition, the entire process of the robot from R&D and manufacturing, assembly and commissioning to transportation, and deployment requires substantial investment of manpower and financial costs. At present, our team is actively conducting communication and collaboration with relevant units, aiming to find a suitable actual pipeline experimental environment to verify various performance indicators of the robot, laying a foundation for subsequent engineering applications.

### 6.2. Experimental Protocol Design

#### 6.2.1. Experimental Verification Objectives

This experiment aims to systematically evaluate the core performance and engineering applicability of the trenchless internal wall grinding robot for large-diameter Prestressed Concrete Cylinder Pipes (PCCP), providing data support for its optimization and engineering application. Specific objectives are as follows:

1. Verify the motion reliability of the robot in real pipeline environments (walking stability, traction performance, obstacle-passing ability, flexibility in curved pipe passage, and positioning accuracy);
2. Evaluate the quality of grinding operations and verify whether the grinding efficiency, flatness, and uniformity meet the requirements;
3. Test the stability and durability of long-term continuous operation, and clarify the endurance capacity and service life of key components;
4. Verify the response timeliness, command accuracy of the remote-control system, and the safety of human-computer interaction.

#### 6.2.2. Experimental Verification Contents and Evaluation Indicators

(1) Motion Performance Verification
  1. Walking performance: Test the maximum forward and reverse walking speeds and uniform speed stability of the robot along the pipeline, and record the jitter amplitude;
  2. Traction performance: Test the maximum weight of heavy objects that the robot can drag while walking and the maximum length of the dragged cable.
  3. Steering performance: Test the steering angle range, curved pipe passage time, and trajectory accuracy in the curved section of the pipeline;
  4. Positioning accuracy: Preset marker points on the pipe wall and test the positioning deviation of the robot.

Evaluation indicators: Maximum walking speed (m/min), uniform speed jitter amplitude (mm), steering response time (s), positioning deviation (mm).

(2) Grinding Operation Quality Verification
  1. Grinding efficiency: Select areas with different rust/attachment thicknesses and test the grinding area per unit time;
  2. Grinding flatness: Use equipment such as a laser range finder to detect the unevenness of the pipe wall after grinding;
  3. Grinding uniformity: Detect the roughness of the ground area and verify whether it meets the preprocessing requirements and uniformity.

Evaluation indicators: Grinding area per unit time (m^2^/min), maximum unevenness (mm), average roughness (μm), roughness standard deviation (μm).

(3) Stability and Durability Verification
  1. Continuous operation stability: Record changes in equipment operating parameters during continuous grinding operations and observe fault conditions;
  2. Durability: Carry out long-term cycle experiments simulating engineering intensity and inspect the wear of key components after the experiment.

Evaluation indicators: Continuous trouble-free operation time (h), wear amount of key components (mm), fluctuation range of operating parameters.

(4) Remote Control Performance Verification
  1. Response timeliness: Test the time difference from the issuance of a control command to its execution;
  2. Control accuracy: Test the consistency of continuous command execution and check for misoperations;
  3. Human-computer interaction convenience: Have operators with different experience levels perform remote control, record the task completion time, and collect evaluation opinions.

Evaluation indicators: Command response time (s), execution accuracy rate (%), average task completion time of operators with different experience levels (min).

### 6.3. Conclusions

(1) A comprehensive analysis was conducted on the influence of the pipeline robot’s attitude angle on traction force. When the robot’s attitude angle γ = 0, the pipe support force is maximized, and the robot’s traction force reaches its peak at this point. Thus, γ = 0 is the optimal attitude angle for the robot.
(2) The traction force of the robot was analyzed under two conditions: no tension and with tension. In the no-tension condition, the robot’s traction force is fully provided by the upper and lower wheel sets; in the tension condition, the traction force is jointly provided by the upper, lower, left, and right wheel sets. Additionally, the spring preload forces required for each wheel set to drive under both conditions were calculated.
(3) In Adams, simulations of the pipeline robot’s traversal, spiral grinding, and straight-elbow pipe traversal were carried out. The robot’s centroid position, velocity, and acceleration were analyzed and measured. The results show that the pipeline robot designed in this paper can smoothly traverse the pipe.
(4) By analyzing the motion trajectories of the four-wheel sets when the robot traverses the elbow, the angular velocity of each wheel set during elbow traversal was planned. Through the analysis of the grinding trajectory of the robot’s three steel brushes during rotation, the condition for the three steel brushes to achieve full coverage of the pipe inner wall during spiral grinding was derived. All the above analyses and verifications were completed in Adams, laying a foundation for the subsequent motion planning and control of the pipeline robot.
(5) A prototype of the large-diameter pipe inner wall grinding robot designed in this paper was fabricated, and its basic functions were debugged. Due to limitations of the experimental conditions, experimental verification of the robot has not yet been carried out; further experiments and optimizations will be conducted in the future.

## Figures and Tables

**Table 1:** Material parameters.

Parameter	Value
Material	Steel
Density/(kg·mm^−3^)	7.801 × 10^−6^
Young’s modulus	2.07 × 10^−5^
Poisson’s ratio	0.29

**Table 2:** Calculation of maximum obstacle-surmounting heights for wheels of different sizes.

Wheel Radius	40 mm	60 mm	80 mm
Maximum Obstacle-surmounting Height	≈10.87 mm	≈16.31 mm	≈21.74 mm

**Table 3:** Summary of simulation experimental results.

**Wheel Radius**	**Theoretical Value**	**Simulation Results**			
40 mm	10.87	8 mm	9 mm	10 mm	11 mm
		**Obstacle-negotiation time span(s)**			
		0.22	0.28	0.38	1.71
**Wheel radius**	**Theoretical value**	**Simulation results**			
60 mm	16.31	14 mm	15 mm	16 mm	17 mm
		**Obstacle-negotiation time span(s)**			
		0.33	0.42	0.53	0.96
**Wheel radius**	**Theoretical value**	**Simulation results**			
80 mm	21.74 mm	19 mm	20 mm	21 mm	22 mm
		**Obstacle-negotiation time span(s)**			
		0.36	0.39	0.45	0.77

## Data Availability

The data presented in this study are available on request from the corresponding author.
